# Asymptotic quantification of entanglement with a single copy

**DOI:** 10.1038/s41567-026-03182-x

**Published:** 2026-03-13

**Authors:** Ludovico Lami, Mario Berta, Bartosz Regula

**Affiliations:** 1 Scuola Normale Superiore, Pisa, Italy; 2 QuSoft, Amsterdam, the Netherlands; 3 Korteweg–de Vries Institute for Mathematics, University of Amsterdam, Amsterdam, the Netherlands; 4 Institute for Theoretical Physics, University of Amsterdam, Amsterdam, the Netherlands; 5 Institute for Quantum Information, RWTH Aachen University, Aachen, Germany; 6 Mathematical Quantum Information RIKEN Hakubi Research Team, RIKEN Pioneering Research Institute (PRI) and RIKEN Center for Quantum Computing (RQC), Wako, Japan

**Keywords:** Quantum information, Information theory and computation

## Abstract

Despite the central importance of quantum entanglement in quantum technologies, understanding the optimal ways to exploit it is still beyond our reach, and even measuring entanglement in an operationally meaningful way is prohibitively difficult. Here we study two fundamental tasks in the processing of entanglement: entanglement testing, which is a quantum state discrimination problem concerned with detecting entanglement in the many-copy regime, and entanglement distillation, which is concerned with purifying entanglement from noisy entangled states. We introduce a way of benchmarking the performance of distillation that focuses on the best achievable error rather than its yield in the asymptotic limit. When the underlying set of operations used for entanglement distillation is the axiomatic class of non-entangling operations, we show that the two figures of merit for entanglement testing and distillation coincide. We solve both problems by proving a generalized quantum Sanov’s theorem, which enables the exact evaluation of the asymptotic error rates of composite quantum hypothesis testing. We show in particular that the asymptotic figure of merit is given by the reverse relative entropy of entanglement, a single-letter quantity that can be evaluated using only a single copy of a quantum state—a distinct feature among measures of entanglement that quantify the optimal performance of information-theoretic tasks.

## Abstract (web summary)

Quantum information theory typically considers the asymptotic limit of having access to many copies of quantum states, which can be challenging to analyse. Now an asymptotic measure of entanglement that only needs a single copy of a quantum state has been found

## Main

Quantum entanglement is one of the most important resources that underlie the potential of quantum technologies to provide advantages in information processing and computation^1–6^. Understanding how to process and use entanglement is crucial to its applications, but our knowledge of the optimal performance of operational tasks involving entanglement is still incomplete. Two key examples of such problems, which turn out to be profoundly connected, are entanglement testing and entanglement distillation.

Entanglement testing can be understood as a type of entanglement detection. In this task, one wishes to certify whether an untrusted source that is supposed to generate copies of some entangled state *ρ*_*AB*_ is performing as intended or, alternatively, is faulty and produces only separable (unentangled) states. Natural figures of merit for this task are based on the minimal probabilities of a misdetection, which could be either a false positive—mistaking a working device for a faulty one—or, vice versa, a false negative. In light of this operational interpretation, any such metric can be interpreted as a measure of the entanglement content of *ρ*_*AB*_: the more entangled the state is, the easier it is to distinguish from separable ones. Such questions are naturally characterized through the framework of composite quantum hypothesis testing^7^, but despite active progress in the study of related problems^8–11^, obtaining a computable expression for optimal performance in this task has been elusive.

Another key operational primitive of quantum information processing is entanglement distillation, a task introduced in the pioneering works of refs. ^12–14^, which aims to purify noisy entangled states into maximally entangled ones. This process is an important ingredient in many practical quantum information protocols, as high-fidelity entanglement is typically a prerequisite for quantum computation and communication schemes. Moreover, entanglement distillation is deeply connected to the theory of quantum error correction^14^. In spite of its importance, and despite it being one of the very first operational protocols ever studied in quantum information theory, we still do not have a complete understanding of entanglement distillation. Most notably, we lack a computable formula for how much entanglement can be distilled from a given quantum state, and even deciding whether any entanglement whatsoever can be extracted is an unsolved problem in general^15,16^. Similar problems affect other entanglement processing tasks, and exact solutions generally exist for only a few special cases.

The main difficulty in studying either of these tasks is that performance can typically be improved by employing more copies of a given quantum state, which means that the ultimate efficiency of a protocol needs to be understood in an asymptotic sense: given more and more copies of a given quantum state, how does the performance improve? This leads to a natural information-theoretic description of such tasks in terms of asymptotic rates, whose evaluation is the main bottleneck for gaining an understanding of the operational properties of quantum entanglement.

An unfortunate consequence of this asymptotic character of entanglement processing is that, even when one can identify a relevant closed-form quantity that describes the given task—such as, for example, quantum relative entropy^17^ or the entanglement of formation^14,18^—the optimal asymptotic rate can be expressed only by using so-called regularized formulas, which require the evaluation of an explicit limit in the number of copies of the given quantum state *ρ*_*AB*_ (refs. ^19–23^). This leads to expressions of the form ${\mathrm{lim}}_{n\to \infty }\frac{1}{n}f({\rho }_{AB}^{\otimes n})$, which are immensely difficult to evaluate, even for simple functions *f*, thus preventing an efficient quantitative characterization of the asymptotic operational properties of entanglement. Because of this, the optimal rates of not only entanglement testing or distillation but also other important operational tasks remain inaccessible in general. When it comes to distillation, this problem persists not merely in the standard, practically motivated setting for manipulating entanglement—namely, in the paradigm of local operations and classical communication (LOCC)^12–14^ —but even in simplified mathematical frameworks where entanglement manipulation is studied under relaxed constraints^24–26^ that are designed to provide a more tractable structure for studying entanglement conversion.

One may then wonder: if precise answers are so hard to find, could we instead obtain insights into the asymptotic properties of quantum entanglement by adjusting the questions that we ask? More specifically, although traditional approaches to entanglement processing remain fundamental and key for many applications, could we obtain a simpler asymptotic characterization of these tasks by changing the way in which we benchmark the performance of protocols, such as by shifting the focus to another figure of merit? This question will motivate the core of our approach.

For entanglement testing, this will entail a seemingly minor change of focus from the asymptotic probability of a false negative (type II error exponent), which is what most previous works have been concerned with^7,8,10,11^, to the asymptotic probability of a false positive (type I error exponent), for which a closed-form solution was unknown before our work^9^. The study of these two deceptively similar variants of the problem requires conceptually different techniques, and—crucially—we will see that this modification will lead to a notable simplification of the resulting expression.

To characterize entanglement distillation, we propose a conceptual shift: instead of focusing on the quantity (yield) as the measure of the efficiency of the protocol when more and more copies of a given state are available, we will focus on the quality of the obtained entanglement, which is represented by the optimal error exponent—the rate at which the error of the protocol can be decreased. This approach is inspired by the information-theoretic characterization of quantum hypothesis testing^27–29^, where this exponent of error probability constitutes the figure of merit. Although any entanglement manipulation framework can be studied through this lens, here we will focus on the one defined by the axiomatic class of non-entangling operations^26^. This useful relaxation of the operationally motivated LOCC framework has been used to shed light on the connections between entanglement theory and thermodynamics^10,11,26,30,31^, and its axiomatic nature means that it can be generalized even to quantum resources beyond entanglement^10,11,31,32^. The simpler structure of these operations will allow us to obtain an exact asymptotic solution.

Our first result establishes an exact equivalence between the performance of the two tasks discussed above, namely entanglement testing (in its standard formulation, under all physically realizable measurements) and entanglement distillation (under non-entangling operations). We show that the error exponent of entanglement distillation equals the exponent of the false positive error in entanglement testing. This connection will allow us to tackle both of these tasks at the same time through an information-theoretic study of the underlying hypothesis testing problem. Indeed, computing the asymptotic exponent of entanglement testing is a generalization of a result in quantum hypothesis testing known as quantum Sanov’s theorem^9,33,34^. However, the much more complicated structure involved in the problem we encounter here means that no known results are sufficiently general to shed any light on it. The problem is also related to the generalized quantum Stein’s lemma^7,10,11^, which has attracted much attention recently, but its distinct structure means that it requires a different approach.

As our main contribution, we then establish a generalized quantum Sanov’s theorem that yields an exact expression for the asymptotic performance of entanglement testing and, as a result, also for the error exponent of entanglement distillation under non-entangling operations. In particular, we show that the exponent is given by a variant of the relative entropy of entanglement, the reverse relative entropy of entanglement^17,35^. A notable aspect of this result is that the quantity can be evaluated exactly—without regularization—on a single copy of the given quantum state, thus circumventing the problems that affect other measures of entanglement connected with practical tasks. Our result thus establishes the reverse relative entropy as a measure of entanglement with a twofold direct meaning while also being computable without having to evaluate a many-copy limit. Altogether, this gives an exact solution to the problem of entanglement testing and provides an alternative way of benchmarking entanglement distillation, and it avoids the seemingly ubiquitous problem of regularized formulas in the quantification of the performance of asymptotic entanglement processing protocols, thus bypassing the resulting bottlenecks.

## Entanglement testing and distillation

### Entanglement testing

In the basic scenario of quantum hypothesis testing (quantum state discrimination), one is tasked with distinguishing between two quantum states *ρ* and *σ* by performing a collective measurement on *n* copies of the unknown state. The probability of mistaking *ρ* for *σ* decays exponentially as 2^−*cn*^, and it is this error exponent *c* that one aims to quantify to understand how fast the distinguishability improves as more copies become available. Notably, in the limit as *n* → *∞*, the error exponent exactly equals the quantum relative entropy $D(\sigma \| \rho )={\rm{T}}{\rm{r}}[\sigma ({\log }_{2}\,\sigma -{\log }_{2}\,\rho )]$ (refs. ^27,28^). It is this result, known as quantum Stein’s lemma, that gives the quantum relative entropy its operational meaning as a measure of the distinguishability of quantum states.

**Fig. 1:**
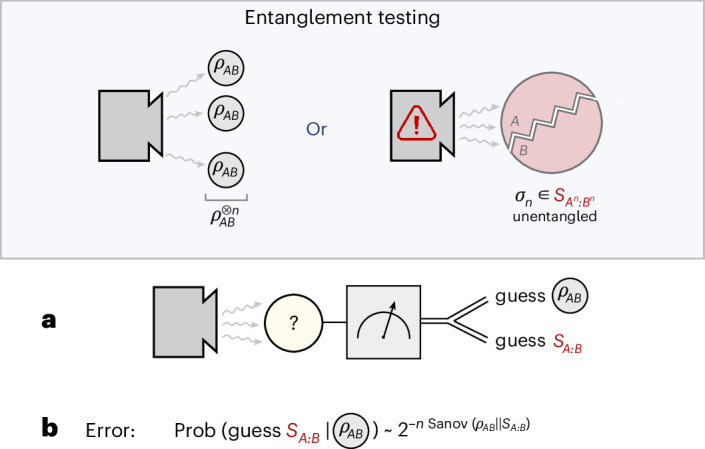
The set-up and figure of merit in entanglement testing. **a**,**b**, Entanglement testing is a quantum hypothesis testing problem concerned with distinguishing the case when a source is generating copies of a target entangled state *ρ*_*AB*_ from the case when it malfunctions and, instead, produces only states ${\sigma }_{n}\in {{\mathcal{S}}}_{{A}^{n}:{B}^{n}}$ that are globally separable, that is exhibit no entanglement between between Alice’s systems on one side and Bob’s systems on the other. **a**, Entanglement testing consists of making a general two-outcome quantum measurement on the overall *n*-copy system that models the output of the device. The choice of the measurement here is arbitrary, and it is the experimenter’s task to optimize this choice. **b**, Two types of error may occur: false positive, where a working device is mistaken for a faulty one, and false negative, where the opposite happens. By choosing a measurement optimally, the probability of a false negative can be constrained to be arbitrarily small while the probability of a false positive can be made to decay exponentially fast to zero. The coefficient governing this exponential behaviour, called the Sanov exponent, is a central object of interest in this work.

Consider now a scenario in which two separated parties, Alice and Bob, would like to use a device that is supposed to prepare *n* copies of some entangled state *ρ*_*AB*_. However, they suspect that the device may fail such that it prepares a state that has no entanglement whatsoever between Alice’s and Bob’s systems. How can we verify whether we have really obtained the desired entangled state? This task, which we call entanglement testing, can be phrased as a composite hypothesis testing problem^7^: we are to distinguish between ${\rho }_{AB}^{\otimes n}$ and the whole set of separable quantum states with a measurement (Fig. 1). Just as in conventional hypothesis testing, we would like to understand the behaviour of the optimal error exponent for large *n*, where the optimization refers to the discrimination strategies. So that this can characterize the optimal performance of the most general discrimination schemes, we do not impose any a priori constraints on the kind of measurement that can be carried out on the system, meaning that the above optimization is understood to run over all physically realizable quantum measurements. This, in turn, makes the error exponent difficult to control and constitutes the main challenge in understanding asymptotic entanglement testing.

There is, however, a certain freedom in choosing which type of error we quantify here. The so-called type I error (false positive) occurs when we mistake ${\rho }_{AB}^{\otimes n}$ for a separable state, whereas a type II error (false negative) occurs when we mistake a separable state for ${\rho }_{AB}^{\otimes n}$. For a fixed, arbitrarily small type I error probability, the asymptotic exponent of the type II error probability is known as the Stein exponent; conversely, the asymptotic exponent of the type I error probability with type II probability fixed (arbitrarily small) is known as the Sanov exponent. The Stein exponent of entanglement testing was first investigated in the works of Brandão and Plenio^7,36^, although it was fully solved only very recently^8,10,11^. Here we will focus instead on the Sanov exponent, which we formally define as

1 $$\begin{array}{l}{\mathrm{Sanov}}({\rho}_{AB}\| {\mathcal{S}}_{A:B}) \\ \quad \, \,\, :\! =\mathop{\rm{lim}}\limits_{\varepsilon \to 0}\mathop{{\mathrm{lim}}\,{\rm{inf}}}\limits_{n\to \infty}-\frac{1}{n}{\log }_{2}\,\min \Big\{{\mathrm{Tr}}\,{M}_{n}{\rho}_{AB}^{\otimes n}\,\Big| 0 \le {M}_{n}\le {\mathbb{1}}, \\ \qquad \qquad \qquad \qquad \qquad \quad \qquad \,\,\,\, {\mathrm{Tr}}({\mathbb{1}}-{M}_{n}){\sigma }_{n}\le \varepsilon \;\,\forall \,{\sigma }_{n}\in {{\mathcal{S}}}_{{A}^{n}:{B}^{n}}\Big\},\end{array}$$

where ${{\mathcal{S}}}_{{A}^{n}:{B}^{n}}$ is the set of all separable states on the *n*-partite quantum system *A*^*n*^*B*^*n*^ that is composed of *n* subsystems *A*^*n*^ = *A*_1_…*A*_*n*_ held by Alice and *n* subsystems *B*^*n*^ = *B*_1_…*B*_*n*_ held by Bob, and where $({M}_{n},{\mathbb{1}}-{M}_{n})$ denotes the positive operator-valued measure (POVM) elements of the measurement performed on the *n*-copy system. Here and elsewhere, $\mathbb{1}$ denotes the identity operator. The evaluation of this exponent will turn out to be closely connected with the task of entanglement distillation.

### Entanglement distillation

The basic setting of entanglement distillation, as introduced in refs. ^12–14^, is as follows. Our protagonists, Alice and Bob, share many copies of a bipartite quantum state *ρ*_*AB*_ and aim to extract pure, maximally entangled states from it. Specifically, they can apply a sequence of quantum channels *Λ*_*n*_, subjected to some locality constraints to be specified later, such that, when acting on *n* copies of *ρ*_*AB*_, the final state approximates *m* copies of the maximally entangled state $| {\varPhi }_{+}\rangle =\frac{1}{\sqrt{2}}(| 00\rangle +| 11\rangle )$, up to an error *ε*_*n*_. We write this as ${\varLambda }_{n}({\rho }_{AB}^{\otimes n}){\approx }_{{\varepsilon }_{n}}| {\varPhi }_{+}\rangle {\langle {\varPhi }_{+}| }^{\otimes m}$, where *ε*_*n*_-closeness is measured by a suitable measure of distance, either the fidelity or, equivalently, the trace distance. Crucially, although the transformation here is approximate and allows for some error, we will require that ${\mathrm{lim}}_{n\to \infty }{\varepsilon }_{n}=0$: as more and more copies of *ρ*_*AB*_ become available, the quality of the distilled entanglement increases, becoming perfect in the asymptotic limit. Now, if we understand *m*/*n* as the yield of this protocol, distillable entanglement *E*_d_(*ρ*_*AB*_) is then defined as the largest asymptotic yield ${\mathrm{lim}}_{n\to \infty }m/n$ optimized over all feasible protocols such that the error *ε*_*n*_ vanishes asymptotically.

Naturally, not all protocols *Λ*_*n*_ can be implemented by two parties that are spatially separated. Therefore, the optimization must be restricted to a suitable class of allowed protocols—often called ‘free operations’—that respect the locality constraints between Alice and Bob. Although the precise choice of the free operations depends on the specific setting under consideration, the most physically natural and commonly adopted class is that of LOCC, as defined originally in refs. ^12–14^. Although well motivated practically, this set is known to have an extremely complicated mathematical structure^37^, which, in particular, hinders one from gaining an understanding of asymptotic entanglement transformations. This has led to a long history of alternative approaches in which one provides extra resources or otherwise extends the allowed operations beyond the LOCC set^24–26,38^, resulting in invaluable insights into the foundations of the theory as well as into the operational power of the LOCC operations themselves. Here we follow these ideas and adopt the axiomatic framework of Brandão and Plenio^26,36^: we consider as free all non-entangling protocols *Λ*_*n*_. That is, all quantum channels that are unable to generate any entanglement: *Λ*_*n*_(*σ*) must remain unentangled for all unentangled states *σ*. This weak requirement is inspired by axiomatic approaches to the second law of thermodynamics^39,40^, and it has already shed light on the theory of entanglement manipulation through these fundamental thermodynamic connections^10,11,26,30,31^. Unlike the LOCC-based approach, the Brandão–Plenio one has the added advantage of being fundamentally resource-agnostic, meaning that it can be extended beyond entanglement and could lead to a unified theory of all quantum resources.

**Fig. 2:**
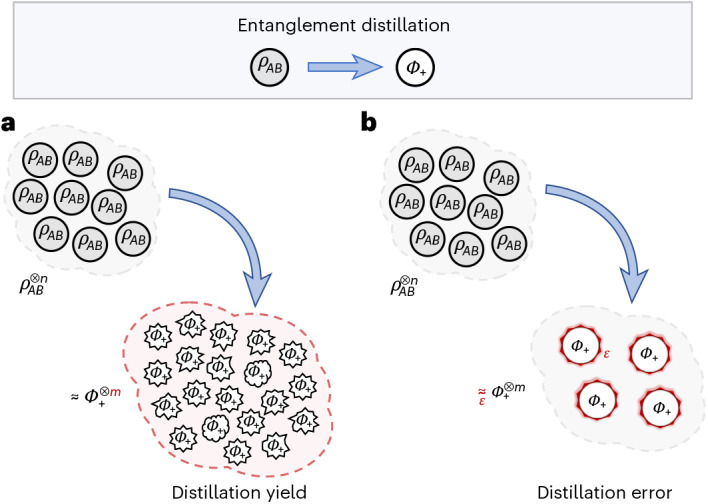
Two ways of benchmarking entanglement distillation. **a**,**b**, Entanglement distillation is the process of converting copies of a noisy entangled quantum state *ρ*_*AB*_ into fewer copies of the pure maximally entangled state *Φ*_+_. To account for physical imperfections in manipulating quantum states, the process is not required to be exact: the resulting states must approximate copies of *Φ*_+_ only to some desired degree of precision, as quantified by the distillation error *ε*. **a**, Conventional approaches to distillation focus on maximizing the distillation yield, that is, the number of copies of *Φ*_+_ obtained for each copy of *ρ*_*AB*_. The error of the procedure is irrelevant as long as it converges to zero in the asymptotic limit as the available number of copies of *ρ*_*AB*_ grows to infinity. For a fixed number of copies, the errors may be large. **b**, In this paper, we instead focus on minimizing the above error, potentially sacrificing some yield to obtain a higher quality entanglement. Specifically, we require that the distillation error vanishes exponentially fast as the number of available copies of *ρ*_*AB*_ grows, while the total number of maximally entangled states *Φ*_+_ produced in the process is still as large as desired. Accordingly, our figure of merit is not the number of copies produced but the optimal error exponent, that is, the rate of decay of the distillation error, which directly quantifies the quality of the entanglement at the output of the protocol.

Taking inspiration from quantum hypothesis testing, where the error exponents are the figures of merit, we can apply similar reasoning here and ask about the distillation error exponent. Specifically, consider again a distillation protocol that outputs *m* copies of $| {\varPhi }_{+}\rangle$ with error *ε*_*n*_. We will now ask: how fast does the quality of the distilled entanglement improve as the number of distilled copies *m* grows to infinity? Instead of focusing on the optimal yield, we will, thus, require that *ε*_*n*_ decay as 2^−*cn*^ and characterize the optimal error exponent *c* (Fig. 2). The distillable entanglement error exponent is then defined as the largest such exponent that can be achieved as the sizes of the input and output systems grow:

2 $$\begin{array}{l}{E}_{{d},{\rm{err}}}(\rho ) \\ \quad :\!=\displaystyle \mathop{\rm{lim}}\limits_{m\to \infty}{\rm{sup}}\left\{\!\mathop{\rm{lim}}\limits_{n\to \infty }-\displaystyle\frac{1}{n}{\log }_{2}{\varepsilon }_{n}{\Big| {\Lambda}_{n} }({\rho }_{AB}^{\otimes n}) {\approx }_{{\varepsilon }_{n}}{\left|{\Phi }_{+}{\rangle}{\langle} {\Phi }_{+}\right|}^{\otimes m}, {\Lambda}_{n}\!\in {\rm{NE}}\,\,{{\forall}}n\right\},\end{array}$$

where we optimize over sequences of non-entangling distillation protocols (NE) to find the least achievable error. Notice that this definition no longer places any importance on the precise number of maximally entangled copies that we obtain in the protocol (provided that it can be made as large as desired) but on only the exponentially decreasing error. This provides an alternative angle for assessing the performance of distillation protocols and is incomparable with previous approaches that focused on the distillation yield.

### Connecting entanglement testing with entanglement distillation

A curious—and very consequential^8,41^—connection between entanglement testing and distillation was shown in the works of Brandão and Plenio^36^, where the Stein exponent of entanglement testing was connected with the asymptotic yield of entanglement distillation in the axiomatic setting of non-entangling operations. Here we establish a dual to that result, one that proves an exact connection between the Sanov exponent and the error of entanglement distillation.

#### Lemma 1

*The asymptotic error exponent of entanglement distillation under non-entangling operations equals the Sanov error exponent in the hypothesis testing of all separable states* ${{\mathcal{S}}}_{A:B}$ *against* *ρ*_*AB*_*:*

3 $${E}_{{\rm{d}},{\rm{e}}{\rm{r}}{\rm{r}}}({\rho }_{AB})={\rm{S}}{\rm{a}}{\rm{n}}{\rm{o}}{\rm{v}}({\rho }_{AB}\| {{\mathcal{S}}}_{A:B}).$$

This shows an equivalence between two a priori rather different tasks: one concerned with extracting entanglement, one with simply detecting it. That the two can be so closely connected will prove extremely useful to us, as we will be able to employ the mathematical machinery of information theory to resolve the asymptotic exponent exactly. We stress that, although the study of entanglement distillation depends on the choice of the allowed free operations (here, non-entangling operations), the task of entanglement testing is defined independently of such constraints; it follows the standard definition of quantum state discrimination, in which all measurements allowed by quantum mechanics are considered.

## A generalized quantum Sanov’s theorem

Just as the exponent of hypothesis testing between two states is given by the quantum relative entropy *D*(*σ*∥*ρ*), it is natural to expect the relative entropy to make an appearance in characterizing the asymptotic exponent of entanglement testing. However, formalizing such connections goes beyond the current state of the art in composite quantum hypothesis testing and would require the development of new techniques.

Our main result is the complete solution of the Sanov exponent of entanglement testing, which by Lemma 1 also gives a resolution of the error exponent of entanglement distillation under non-entangling operations. The key role here will be played by the reverse relative entropy of entanglement, defined as^35^

4 $$D({{\mathcal{S}}}_{{\rm{A}}:{\rm{B}}}\| {\rho }_{\mathrm{AB}})\,:\! =\mathop{\min }\limits_{{\sigma }_{\mathrm{AB}}\in {{\mathcal{S}}}_{{\rm{A}}:{\rm{B}}}}\,D({\sigma }_{\mathrm{AB}}\| {\rho }_{\mathrm{AB}}),$$

where the term ‘reverse’ refers to the fact that the relative entropy of entanglement was originally defined with the arguments in the opposite order, as $D({\rho }_{\mathrm{AB}}\| {{\mathcal{S}}}_{{\rm{A}}:{\rm{B}}})\,:\!=\mathop{\min }\limits_{{\sigma }_{\mathrm{AB}}\in {{\mathcal{S}}}_{{\rm{A}}:{\rm{B}}}}\,D({\rho }_{\mathrm{AB}}\| {\sigma }_{\mathrm{AB}})$ (ref. ^17^).

### Theorem 2

*For any state ρ*_*AB*_ *, the asymptotic Sanov error exponent of entanglement testing under all physical quantum measurements—and, as a result, the error exponent of entanglement distillation under non-entangling operations—equals the reverse relative entropy of entanglement:*

5 $${\rm{S}}{\rm{a}}{\rm{n}}{\rm{o}}{\rm{v}}({\rho }_{AB}\| {{\mathcal{S}}}_{A:B})=D({{\mathcal{S}}}_{A:B}\| {\rho }_{AB})={E}_{{\rm{d}},{\rm{e}}{\rm{r}}{\rm{r}}}({\rho }_{AB}).$$

A notable aspect of the result is that, although both the distillable entanglement error exponent *E*_d,err_ and the Sanov exponent express asymptotic information-theoretic properties of the quantum state *ρ*_*AB*_—they quantify the performance of ${\rho }_{AB}^{\otimes n}$ in the limit of large *n*—the quantity $D({{\mathcal{S}}}_{A:B}\| {\rho }_{AB})$ is single letter, in that it does not require a regularization and can be evaluated on a single copy of *ρ*_*AB*_. This lets us avoid the biggest issue that plagues most solutions for the asymptotic rates of entanglement manipulation protocols.

The main technical hurdle in proving Theorem 2 is that the hypotheses (states) involved in the discrimination task depart from the typically considered setting of independent and identically distributed (i.i.d.) ones. In recent years, there has been significant interest in such hypothesis testing tasks ‘beyond i.i.d.’ in quantum information theory^7,8,10,11,33,34,42–47^, but none of the previous results are sufficiently general to cover our setting. Our proof of the theorem proceeds in two steps. First, we prove the corresponding result in classical information theory, where, instead of quantum states, we constrain ourselves to classical probability distributions. Despite the mathematically simpler structure, this result is already non-trivial, as some intuitive approaches used in i.i.d. cases fail to work. Instead, we employ a recently introduced powerful mathematical technique called blurring^11^, which allows us to handle general composite problems in hypothesis testing. Finally, we show that the classical solution can be lifted to a fully quantum one by performing suitable measurements on the quantum systems under consideration. Our solution of the problem is, in fact, very general and extends beyond entanglement testing to the testing of more general quantum resources. The intuition for the proof method is presented in Methods, and the complete proof can be found in Supplementary Information.

Although conceptually somewhat different, our result may be compared with previous related findings that evaluated asymptotic rates of entanglement distillation by connecting them with hypothesis testing problems. This notably includes the generalized quantum Stein’s lemma, as originally conjectured in ref. ^7^ and recently proven in refs. ^10,11^. The result states that the Stein exponent of entanglement testing—the asymptotic exponent of the type II error probability in discriminating a given state $${\rho }_{AB}^{\otimes n}$$ from all separable states—is given exactly by the regularized relative entropy of entanglement, $${D}^{\infty }({\rho }_{AB}\| {{\mathcal{S}}}_{A:B})$$$$\,:={\mathrm{lim}}_{n\to \infty }\frac{1}{n}D({\rho }_{AB}^{\otimes n}\| {{\mathcal{S}}}_{{A}^{n}:{B}^{n}})$$. As shown in ref. ^36^, this also equals the asymptotic yield of entanglement distillation under non-entangling operations. The main difference between this result and ours is the need for regularization: although the generalized quantum Stein’s lemma ostensibly provides an exact expression of the distillable entanglement, this is given by a regularized quantity, which prevents an efficient evaluation of it except for limited special cases. A variant of this result was also shown in a setting less permissive than all non-entangling operations, namely, the more restricted class of ‘dually non-entangling operations’ ^48^, where the entanglement yield can again be evaluated through a connection with a composite hypothesis testing problem^42^. This asymptotic rate is, however, also affected by the problem of regularization, which our result in Theorem 2 completely sidesteps.

Some words on the applicability of Theorem 2 are in order. There are instances of quantum states from which maximal entanglement can be distilled exactly, with no error. This notably includes pure entangled states ${\rho }_{AB}=| {\psi }_{AB}\rangle \langle {\psi }_{AB}|$ (ref. ^49^). As a consequence, in such cases the error exponent can be chosen to be arbitrarily high, and so *E*_d,err_ diverges to infinity. This is, indeed, expected and is fully captured by Theorem 2: for all such states, we have that $D({{\mathcal{S}}}_{A:B}\| {\rho }_{AB})=\infty$. Although this looks as if it may limit the applicability of our result, such cases highly contrast with quantum states typically encountered in experimental settings: perfect zero-error entanglement extraction is impossible from all full- or high-rank quantum states^50,51^, meaning that $D({{\mathcal{S}}}_{A:B}\| {\rho }_{AB})$ is necessarily finite for all generic *ρ*_*AB*_. Computing the asymptotic rates of entanglement distillation has been a difficult task for these noisy states, as conventional techniques in entanglement distillation, which can provide a complete description of distillation for pure states^12^, have not managed to shed much light on the general case of mixed states. This means that our results could find direct applicability as an entanglement benchmark in the regime complementary to the well-studied and well-understood setting of noiseless pure states by serving as a well-behaved entangled measure for generic noisy quantum systems.

## Discussion

The main significance of our result is the demonstration that truly asymptotic properties of entanglement can be characterized exactly without the need to consider asymptotic and regularized entanglement measures. This is important from a computational perspective—as evaluating regularized quantities is typically extremely hard, making it difficult to quantify optimal rates and give benchmarks on feasible protocols—but also from a theoretical one, as single-letter expressions are much easier to characterize mathematically and can lead to an improved theoretical understanding of the ultimate limitations of entanglement manipulation.

Our findings also strengthen the connections between the theories of entanglement testing and axiomatic entanglement distillation by giving a twofold meaning to the reverse relative entropy of entanglement $D({{\mathcal{S}}}_{A:B}\| {\rho }_{AB})$, an entropic entanglement measure, being both the optimal rate of type I error in entanglement testing and being the error exponent of entanglement distillation under non-entangling operations.

These developments all rest on our key technical result, the generalized quantum Sanov’s theorem, which allows us to characterize quantum hypothesis testing tasks where one of the hypotheses is very general; in entanglement theory, it is the whole set of separable states. The result represents an advance in the theory of quantum hypothesis testing, as dealing with such non-i.i.d. hypotheses has long been a main obstacle. Indeed, a gap in the original proof of the generalized quantum Stein’s lemma was found^8,41^, one that stemmed from the difficulty in composite hypothesis testing; only recently have complete solutions finally appeared^10,11^, and one of these techniques—namely, the blurring method introduced in ref. ^11^—has allowed us to resolve the generalized quantum Sanov’s theorem. As we discuss in more detail in Supplementary Note D, the close relation between the reverse relative entropy and quantum hypothesis testing can be extended beyond the theory of quantum entanglement to more general sets of quantum states. Developing the technical methods needed to handle such composite, non-i.i.d. problems remains one of the main open problems of quantum information theory.

Our evaluation of the error exponent of entanglement distillation, on the other hand, provides an alternative angle that is not directly comparable with the original frameworks of entanglement distillation based on asymptotic yield^14^. Nevertheless, it is worth noting that in the latter settings, single-letter solutions were known only in very limited special cases, for example pure^12^ or maximally correlated states^24^. Additionally, simplified and computable asymptotic solutions can sometimes be obtained in ‘zero-error’ entanglement manipulation^38,52^, where one imposes that no error can be made whatsoever; such settings are, however, highly idealized and not directly useful in practice. To the best of our knowledge, our work represents the first solution of an asymptotic entanglement transformation protocol, in the sense of an asymptotic task with error vanishing in the limit, that admits a single-letter solution for all quantum states.

The precise connection with entanglement distillation here relies on the choice of the axiomatic framework of non-entangling operations. Although often considered simpler, such axiomatic approaches have not previously been shown to lead to single-letter expressions in the asymptotic study of entanglement. It would certainly be interesting to extend this relation to other sets of free operations, but such an exact correspondence is most probably impossible in the most practical settings such as LOCC due to the difficulties of characterizing bound entanglement^15^. The advantage of the axiomatic approach that we have shown is that it allows for these deep connections, both conceptual and quantitative, to be established. Importantly, however, the equality between the reverse relative entropy and the exponent of entanglement testing is completely independent of our assumptions on axiomatic entanglement distillation: indeed, entanglement testing does not hinge on any choice of free operations and uses only the basic structure of quantum measurements and separable states.

A conclusion that one may draw from our approach is that, when dealing with asymptotic protocols, it can be beneficial to change one’s way of looking at the problem by focusing on the error exponent rather than the asymptotic yield. This seemingly simple insight opened the door to major developments in our understanding of entanglement manipulation: it is what allowed us to establish the connection between entanglement distillation and quantum Sanov’s theorem in entanglement testing, ultimately leading to a complete single-letter solution of the distillable entanglement error exponent. The basic idea can be immediately generalized to a myriad of other settings in quantum and classical information, and we hope that this will lead to many more fruitful connections and developments in quantum information processing.

## Methods

The aim of this section is to provide intuition for the main technical contributions of our approach as well as the main difficulties we had to avoid on the way to establishing a generalized quantum Sanov’s theorem, together with its equivalence with the exponent of entanglement distillation under non-entangling operations. Full technical proofs can be found in Supplementary Notes A–E.

### Equivalence between entanglement distillation and entanglement testing

Recall that our main object of study is the Sanov exponent ${\rm{S}}{\rm{a}}{\rm{n}}{\rm{o}}{\rm{v}}({\rho }_{AB}\| {{\mathcal{S}}}_{A:B})$. To express this exponent in a convenient way, we will use the hypothesis testing relative entropy^53,54^

6 $${D}_{{\rm{H}}}^{\varepsilon }(\sigma \| \rho )\,:=-{\log }_{2}\,\min \{{\rm{T}}{\rm{r}}\,M\rho \,|\,0\le M\le {\mathbb{1}},\,{\rm{T}}{\rm{r}}({\mathbb{1}}-M)\sigma \le \varepsilon \}.$$

By thinking of *M* as an arbitrary measurement operator—an element of a POVM—we can understand $(M,{\mathbb{1}}-M)$ as the most general two-outcome measurement that we may use to discriminate between *ρ* and *σ*. Assigning the first outcome of this measurement to the state *σ* and the second to *ρ*, ${D}_{{\rm{H}}}^{\varepsilon }(\sigma \parallel \rho )$ then precisely quantifies the optimal type I error exponent of hypothesis testing when the type II error probability is constrained to be at most *ε*. We can then write

7 $${\rm{S}}{\rm{a}}{\rm{n}}{\rm{o}}{\rm{v}}({\rho }_{AB}\| {{\mathcal{S}}}_{A:B}):=\mathop{\mathrm{lim}}\limits_{\varepsilon \to 0}\mathop{{\rm{l}}{\rm{i}}{\rm{m}}\,{\rm{i}}{\rm{n}}{\rm{f}}}\limits_{n\to \infty }\frac{1}{n}{D}_{{\rm{H}}}^{\varepsilon }\big({{\mathcal{S}}}_{{A}^{n}:{B}^{n}}\|\,{\rho }_{AB}^{\otimes n}\big),$$

where we note that the optimized hypothesis testing relative entropy can be written as

8 $$\begin{array}{rcl}{D}_{{\rm{H}}}^{\varepsilon }({{\mathcal{S}}}_{A:B}\| {\rho }_{AB}) & = & \mathop{\min }\limits_{\sigma \in {{\mathcal{S}}}_{A:B}}{D}_{{\rm{H}}}^{\varepsilon }({\sigma }_{AB}\| {\rho }_{AB})\\ & = & -{\log }_{2}\,\min \{{\rm{T}}{\rm{r}}M{\rho }_{AB}\,|\,0\!\le\! M\!\le\! {\mathbb{1}},\,{\rm{T}}{\rm{r}}\,M\sigma \!\ge\! 1\!-\!\varepsilon\ {\rm{\forall }}\,\sigma \in {{\mathcal{S}}}_{A:B}\},\end{array}$$

with the equality on the second line following from von Neumann’s minimax theorem^55^.

We remark here that the name ‘Sanov’s theorem’ is typically used in the classical information theory literature to refer to a slightly different result on the probability of observing samples whose empirical distribution (type) lies in a given set of distributions (Section 11.4 of ref. ^56^). We follow other works in quantum information theory, starting with ref. ^33^, which used the name ‘quantum Sanov’s theorem’ to refer to a hypothesis testing problem with a composite null hypothesis, like (but not directly comparable with) the setting we study here. We specifically use the name ‘generalized quantum Sanov’s theorem’ because our composite hypothesis testing problem involves a general set of non-i.i.d. states ${{\mathcal{S}}}_{{A}^{n}:{B}^{n}}$, in the same way that the ‘generalized quantum Stein’s lemma’ is now commonly used to refer to the closely related composite setting introduced in ref. ^7^. The same generalized variant of quantum Sanov’s theorem was previously studied in ref. ^9^, where only bounds on the optimal asymptotic exponent were obtained (see also section ‘On entropies and their (non-)additivity’). Yet another quantum variant of Sanov’s theorem, more closely related to the original formulation of classical Sanov’s theorem based on empirical distributions, was recently proposed by Hayashi^57^; this, however, is not directly related to the setting studied here.

The claim of our Lemma 1 is the asymptotic equivalence between this quantity and the error exponent of entanglement distillation, which we recall to be

9 $${E}_{{\rm{d}},{\rm{e}}{\rm{r}}{\rm{r}}}({\rho }_{AB})=\mathop{\mathrm{lim}}\limits_{m\to \infty }{E}_{{\rm{d}},{\rm{e}}{\rm{r}}{\rm{r}}}^{(m)}({\rho }_{AB}),$$

where ${E}_{{\rm{d}},{\rm{e}}{\rm{r}}{\rm{r}}}^{(m)}$ denotes the exponent of distillation under non-entangling operations for a fixed number of *m* output copies:

10 $${E}_{{\rm{d}},{\rm{e}}{\rm{r}}{\rm{r}}}^{(m)}({\rho }_{AB}):=\sup \Big\{\mathop{{\rm{l}}{\rm{i}}{\rm{m}}\,{\rm{i}}{\rm{n}}{\rm{f}}}\limits_{n\to \infty }-\frac{1}{n}{\log }_{2}\,{\varepsilon }_{n}\,\Big|\, {{{\Lambda }}}_{n}(\rho^{\otimes n}_{AB}){\approx }_{{\varepsilon }_{n}}| {\varPhi }_{+}\rangle {\langle {\varPhi }_{+}| }^{\otimes m},\,{\varLambda }_{n}\in {\rm{N}}{\rm{E}}\Big\},$$

where the supremum is understood to be over all sequences ${({\varLambda }_{n})}_{n\in {{{\mathbb{N}}}}}$ of operations satisfying the specified constraints and, in particular, belonging to the class of non-entangling maps.

We now outline the main part of our argument, the details of which can be found in Supplementary Note A. The approach bears some technical similarity with a construction used in ref. ^36^, but a crucial difference is that we employ the connection in a rather different way. Instead of the type II hypothesis-testing error, which was the object of study in ref. ^36^, we are interested in the type I error, and suitable modifications of the proof have to be made to account for this. This shift is what distinguishes our approach and ultimately leads to quantitatively different results.

On the one hand, any distillation protocol $${({\varLambda }_{n})}_{n}$$ can be turned into a suitable sequence of tests $$({M}_{n},{\mathbb{1}}-{M}_{n})$$ that perform entanglement testing with a small type I error probability. Because $${\varLambda }_{n}({\rho }_{AB}^{\otimes n})$$$${\approx }_{{\varepsilon }_{n}} |{\varPhi }_{+}\rangle {\langle {\varPhi }_{+}| }^{\otimes m}$$, we can construct a measurement by defining $${M}_{n}\,:={\mathbb{1}}-{\varLambda }_{n}^{\dagger }(| {\varPhi }_{+}\rangle {\langle {\varPhi }_{+}| }^{\otimes m})$$, where $${\varLambda }_{n}^{\dagger }$$ denotes the adjoint map of *Λ*_*n*_. This represents the action of the channel in the Heisenberg picture. We can then show that the type II error probability of this test is at most 2^−*m*^, whereas the type I error is at most *ε*_*n*_; this gives a feasible protocol for entanglement testing, leading to the bound

11 $$\mathop{\min }\limits_{{\sigma }_{n}\in {{\mathcal{S}}}_{{A}^{n}:{B}^{n}}}{D}_{{\rm{H}}}^{{2}^{-m}}\big({\sigma }_{n}\, \|\, {\rho }_{AB}^{\otimes n}\big)\ge {\log }_{2}\frac{1}{{\rm{T}}{\rm{r}}\,{M}_{n}{\rho }_{AB}^{\otimes n}}\ge -{\log }_{2}{\varepsilon }_{n},$$

which is one direction of the claimed relation.

For the other direction, we take any sequence of feasible measurement operators *M*_*n*_ for entanglement testing of ${\rho }_{AB}^{\otimes n}$ and use them to construct a distillation protocol. This is done through a simple measure-and-prepare procedure: we first perform the measurement $({M}_{n},{\mathbb{1}}-{M}_{n})$, and if we obtain the first outcome (we think that the input state is separable), then we simply prepare a suitable separable state; if, however, we obtain the second outcome (we think that the state is ${\rho }_{AB}^{\otimes n}$), then we prepare our desired target state ${| {\varPhi }_{+}\rangle }^{\otimes m}$. In Supplementary Note A we show that this constitutes a feasible distillation protocol with error ${\varepsilon }_{n}=\mathrm{Tr}\,{M}_{n}{\rho }_{AB}^{\otimes n}$, giving

12 $${E}_{{\rm{d}},{\rm{e}}{\rm{r}}{\rm{r}}}^{(m)}({\rho }_{AB})\ge \mathop{{\rm{l}}{\rm{i}}{\rm{m}}\,{\rm{i}}{\rm{n}}{\rm{f}}}\limits_{n\to \infty }\frac{1}{n}\mathop{\min }\limits_{{\sigma }_{n}\in {{\mathcal{S}}}_{{A}^{n}:{B}^{n}}}{D}_{{\rm{H}}}^{{2}^{-m}}\big({\sigma }_{n}\,\|\, {\rho }_{AB}^{\otimes n}\big).$$

Altogether the above arguments show that

13 $$\begin{array}{cl}{E}_{{\rm{d}},\mathrm{err}}({\rho }_{AB}) & =\mathop{\mathrm{lim}}\limits_{m\to \infty }{E}_{{\rm{d}},\mathrm{err}}^{(m)}(\rho )=\mathop{\mathrm{lim}}\limits_{m\to \infty }\mathop{\mathrm{lim}\,\inf }\limits_{n\to \infty }\displaystyle\frac{1}{n}\mathop{\min }\limits_{{\sigma }_{n}\in {{\mathcal{S}}}_{{A}^{n}:{B}^{n}}}{D}_{{\rm{H}}}^{{2}^{-m}}\big({\sigma }_{n}\,\|\, {\rho }_{AB}^{\otimes n}\big)\\ & =\mathrm{Sanov}({\rho }_{AB}\| {{\mathcal{S}}}_{A:B}),\end{array}$$

which establishes an equivalence between the error exponent of entanglement distillation and the Sanov exponent of entanglement testing.

### On entropies and their (non-)additivity

Let us now consider the claim of our main result, namely, that ${\rm{S}}{\rm{a}}{\rm{n}}{\rm{o}}{\rm{v}}({\rho }_{AB}\| {{\mathcal{S}}}_{A:B})=D({{\mathcal{S}}}_{A:B}\| {\rho }_{AB})$.

A simple but key observation that helps motivate this claim is that the reverse relative entropy of entanglement is, in fact, additive on tensor product states^35^. That is, we have

14 $$D({{\mathcal{S}}}_{A{A}^{{\prime} }:B{B}^{{\prime} }}\| {\rho }_{AB}\otimes {\omega }_{{A}^{{\prime} }{B}^{{\prime} }})=D({{\mathcal{S}}}_{A:B}\| {\rho }_{AB})+D({{\mathcal{S}}}_{{A}^{{\prime} }:{B}^{{\prime} }}\| {\rho }_{{A}^{{\prime} }{B}^{{\prime} }})$$

for all states *ρ*_*AB*_ and $${\omega }_{{A}^{{\prime} }{B}^{{\prime} }}$$. To see this, let $${\sigma }_{A{A}^{{\prime} }B{B}^{{\prime} }}\in {{\mathcal{S}}}_{A{A}^{{\prime} }:B{B}^{{\prime} }}$$ be a minimizer of $$D({{\mathcal{S}}}_{A{A}^{{\prime} }:B{B}^{{\prime} }}\| {\rho }_{AB}\otimes {\omega }_{{A}^{{\prime} }{B}^{{\prime} }})$$, and use that $${\log }_{2}({\rho }_{AB}\otimes {\omega }_{{A}^{{\prime} }{B}^{{\prime} }})$$$$={\log }_{2}\,{\rho }_{AB}\otimes {{\mathbb{1}}}_{A'B'}$$$$+{{\mathbb{1}}}_{AB}\otimes {\log }_{2}\,{\omega }_{A'B'}$$ to get

15 $$\begin{array}{cl}D({\sigma }_{A{A}^{{\prime} }B{B}^{{\prime} }}\| {\rho }_{AB}\otimes {\omega }_{{A}^{{\prime} }{B}^{{\prime} }}) & =-S({\sigma }_{A{A}^{{\prime} }B{B}^{{\prime} }})+D({\sigma }_{AB}\| {\rho }_{AB})\\ & \quad\; +D({\sigma }_{{A}^{{\prime} }{B}^{{\prime} }}\| {\omega }_{AB})+S({\sigma }_{AB})+S({\sigma }_{{A}^{{\prime} }{B}^{{\prime} }})\\ & =I{(A{A}^{{\prime} }:B{B}^{{\prime} })}_{\sigma }+D({\sigma }_{AB}\| {\rho }_{AB})+D({\sigma }_{{A}^{{\prime} }{B}^{{\prime} }}\| {\omega }_{AB})\\ & \ge D({{\mathcal{S}}}_{A:B}\| {\rho }_{AB})+D({{\mathcal{S}}}_{{A}^{{\prime} }:{B}^{{\prime} }}\| {\omega }_{{A}^{{\prime} }{B}^{{\prime} }}),\end{array}$$

where the last line follows from the non-negativity of the quantum mutual information $I{(A{A}^{{\prime} }:B{B}^{{\prime} })}_{\sigma }=S({\sigma }_{AB})+S({\sigma }_{{A}^{{\prime} }{B}^{{\prime} }})-S({\sigma }_{A{A}^{{\prime} }B{B}^{{\prime} }})$ (Theorem 11.6.1 in ref. ^58^) and the fact that the reduced systems *σ*_*AB*_ and ${\sigma }_{{A}^{{\prime} }{B}^{{\prime} }}$ are always separable for ${\sigma }_{A{A}^{{\prime} }B{B}^{{\prime} }}$ separable between $A{A}^{{\prime} }$ versus $B{B}^{{\prime} }$. This already tells us that this quantity can help us avoid issues with many-copy formulas, as regularization is simply not needed for this formula.

Although the converse direction ${\rm{S}}{\rm{a}}{\rm{n}}{\rm{o}}{\rm{v}}({\rho }_{AB}\| {{\mathcal{S}}}_{A:B})\le D({{\mathcal{S}}}_{A:B}\| {\rho }_{AB})$ can straightforwardly be concluded from the converse of the standard i.i.d. setting, for the other direction, we need to construct a composite hypothesis test that works well enough to distinguish any separable state from ${\rho }_{AB}^{\otimes n}$. Now, consider first a simpler case: if we were to test against a fixed tensor product state ${\sigma }_{AB}^{\otimes n}$ instead of the whole set of separable states, the quantum Stein’s lemma^27^ would immediately tell us that *D*(*σ*_*AB*_∥*ρ*_*AB*_) is an achievable error exponent. In more detail, the modern and arguably simplest approach for proving the achievability part of i.i.d. quantum hypothesis testing goes through the family of Petz–Rényi divergences${D}_{\alpha }(\sigma \| \rho )=\frac{1}{\alpha -1}{\log }_{2}\,{\rm{T}}{\rm{r}}\,{\sigma }^{\alpha }{\rho }^{1-\alpha }$ (ref. ^59^), which leads via Audenaert et al.’s inequality^60^ to

16 $${\rm{S}}{\rm{a}}{\rm{n}}{\rm{o}}{\rm{v}}({\rho }_{AB}\| {\sigma }_{AB})\ge \displaystyle \mathop{\mathrm{lim}}\limits_{\alpha \to {1}^{-}}\mathop{\mathrm{lim}}\limits_{n\to \infty }\frac{1}{n}{D}_{\alpha }\big({\sigma }_{AB}^{\otimes n}\,\|\, {\rho }_{AB}^{\otimes n}\big)=D({\sigma }_{AB}\| {\rho }_{AB}).$$

Here the crucial point in the derivation is that $${D}_{\alpha }\big({\sigma }_{AB}^{\otimes n}\,\big\| \,{\rho }_{AB}^{\otimes n}\big)$$$$=n{D}_{\alpha }({\sigma }_{AB}\|\, {\rho }_{AB})$$ is an additive bound on the error probability that becomes asymptotically tight with $${\mathrm{lim}}_{\alpha \to {1}^{-}}{D}_{\alpha }({\sigma }_{AB}\|\, {\rho }_{AB})$$$$=D({\sigma }_{AB}\| \,{\rho }_{AB})$$ (ref. ^59^). One might then wonder whether these state-of-the-art quantum hypothesis-testing methods could also be used for the generalized Sanov’s theorem, where the fixed state $${\sigma }_{AB}^{\otimes n}$$ is replaced with the set of states $${{\mathcal{S}}}_{A{:}B}$$.

Indeed, this approach was recently initiated in ref. ^9^, and consequently, the question was raised whether the corresponding Petz–Rényi divergences of entanglement ${D}_{\alpha }({{\mathcal{S}}}_{A:B}\|\, {\rho }_{AB})$ become additive. Perhaps surprisingly, however, we can show that, in contrast to the aforementioned special case *α* = 1, the divergences are not additive for *α* ∈ (0, 1). Namely, by taking the antisymmetric Werner state *ρ*_a_ as an example, it can be shown that^61^ (Supplementary Note E)

17 $${D}_{\alpha }({{\mathcal{S}}}_{A{A}^{{\prime} }:B{B}^{{\prime} }}\| {\rho }_{{\rm{a}}}\otimes {\rho }_{{\rm{a}}}) < 2{D}_{\alpha }({{\mathcal{S}}}_{A:B}\| {\rho }_{{\rm{a}}}).$$

This non-additivity means that, to characterize the generalized Sanov exponent, we would really need to work with the regularized quantities ${\mathrm{lim}}_{n\to \infty }\frac{1}{n}{D}_{\alpha }\big({{\mathcal{S}}}_{{A}^{n}:{B}^{n}}\,\|\, {\rho }_{AB}^{\otimes n}\big)$. Unfortunately, this prevents us from being able to use the known continuity results for the Petz–Rényi divergences (cf. refs. ^8,9^) and makes it difficult to follow the approach of ref. ^9^ to establish a connection with the reverse relative entropy $D({{\mathcal{S}}}_{A:B}\| {\rho }_{AB})$, which is our goal. As such, we need to overcome this technical bottleneck in known proof techniques and develop an approach that will allow us to resolve the generalized Sanov’s theorem.

### Axiomatic approach

Recall that our main goal is to characterize the asymptotic error exponent in entanglement testing, that is, distinguishing a sequence of states ${\rho }_{AB}^{\otimes n}$ from the set of separable states ${{\mathcal{S}}}_{A:B}$. However, it will be useful to forget about separable states for now and try to understand the set in an axiomatic manner, using only some of its basic properties. Such an axiomatic approach is due to the influential works of Brandão and Plenio^7^ in connection with the generalized quantum Stein’s lemma (cf. the recent works in refs. ^8,10,11^).

This has a dual purpose: on the one hand, it will immediately allow us to apply many of our results to quantum resource theories beyond entanglement^32,62^; more importantly, however, it will actually also be a crucial ingredient in our proof of the generalized quantum Sanov’s theorem for entanglement theory itself.

To do this, let us work out a list of abstract mathematical properties obeyed by the set of separable states as well as by other relevant sets of free states. The first five of these properties were proposed by Brandão and Plenio^7^ and are sometimes known as the Brandão–Plenio axioms. To state them, we consider some quantum system with Hilbert space ${\mathcal{H}}$ and a sequence ${({{\mathcal{F}}}_{n})}_{n}$ of sets ${{\mathcal{F}}}_{n}\subseteq {\mathcal{D}}({{\mathcal{H}}}^{\otimes n})$ of density operators on *n* copies of ${\mathcal{H}}$. States in ${{\mathcal{F}}}_{n}$ are conventionally referred to as free states, and a state that is not free is called resourceful. We posit the following axioms:

1. For each *n*, ${{\mathcal{F}}}_{n}$ is a convex and closed subset of states.
2. ${{\mathcal{F}}}_{1}$ contains some full-rank state *σ*_0_ > 0, for example, the maximally mixed state.
3. The family ${({{\mathcal{F}}}_{n})}_{n}$ is closed under partial traces: tracing out any number of the *n* subsystems cannot make a free state resourceful.
4. The family ${({{\mathcal{F}}}_{n})}_{n}$ is closed under tensor products: the tensor product of any two free states is also free.
5. Each ${{\mathcal{F}}}_{n}$ is closed under permutations: permuting any of the *n* subsystems cannot create a resource from a free state.

Picking ${\mathcal{H}}={{\mathcal{H}}}_{AB}={{\mathcal{H}}}_{A}\otimes {{\mathcal{H}}}_{B}$ as a bipartite Hilbert space and taking ${{\mathcal{F}}}_{n}={{\mathcal{S}}}_{{A}^{n}:{B}^{n}}$ as the set of separable states on ${{\mathcal{H}}}_{A}^{\otimes n}\otimes {{\mathcal{H}}}_{B}^{\otimes n}$ (with all *A* systems on one side and all *B* systems on the other) clearly satisfies all of the above Axioms 1–5. However, these axioms are also obeyed by many other sets of free states, corresponding to different quantum resource theories^62^. All of our definitions can be immediately extended to such sets, with the conjectured generalized Sanov’s theorem now asking whether

18 $${\rm{S}}{\rm{a}}{\rm{n}}{\rm{o}}{\rm{v}}(\rho \| {\mathcal{F}})\mathop{=}\limits^{?}D({\mathcal{F}}\| \rho )=\mathop{\min }\limits_{\sigma \in {{\mathcal{F}}}_{1}}D(\sigma \| \rho )\,.$$

Although the above natural set of axioms, indeed, turns out to be sufficient to prove the generalized quantum Stein’s lemma^10,11^, note that the axioms are not sufficient for the generalized Sanov’s theorem. In Supplementary Note E we give a classical example that fulfils Axioms 1–5, while anyway having

19 $${\rm{S}}{\rm{a}}{\rm{n}}{\rm{o}}{\rm{v}}(\rho \| {\mathcal{F}})=0 < \infty =D({\mathcal{F}}\| \rho )$$

for some (classical) state *ρ*. To remedy this problem, we need to introduce a further assumption about the sets ${{\mathcal{F}}}_{n}$. We first consider the following extra axiom:

6. The *regularized relative entropy of resource* is faithful. That is, for all resourceful $$\rho \in {\mathcal{D}}({\mathcal{H}})$$ with $$\rho \notin {{\mathcal{F}}}_{1}$$, we have that $${D}^{\infty }(\rho \,\| \,{\mathcal{F}})$$$$:\!={\mathrm{lim}}_{n\to \infty }\frac{1}{n}\,D({\rho }^{\otimes n}\,\| \,{{\mathcal{F}}}_{n}) > 0$$.

We note here that this concerns the conventional definition of the relative entropy ${D}^{\infty }(\rho \| {\mathcal{F}})$ rather than the ‘reverse’ variant $D({\mathcal{F}}\| \rho )$. This rather non-trivial property is obeyed by many quantum resources encountered in practice. For instance, for separable states, it has been proved to hold independently by Brandão and Plenio (Corollary II.2 in ref. ^7^) and by Piani^63^. It is, however, not universal, and the counterexample in equation (19) violates this axiom. Indeed, Axiom 6 turns out to be sufficient, together with Axioms 1–5, to imply the generalized Sanov theorem in the *fully classical* case. That is, instead of general quantum states, we restrict ourselves to classical probability distributions (commuting states). However, the axiom does not seem to suffice to establish the quantum extension of this finding. To derive the quantum result, we will, instead, need an axiom that is seemingly rather different from Axiom 6 but actually closely related to it. This new Axiom 6’ is concerned with how measurements close to the identity act on the set of free states:

- 6′. For some choice of numbers ${r}_{n}\in (0,1]$, the sequence ${({{\mathbb{M}}}_{n})}_{n}$ of sets of measurements
  20 $${{\mathbb{M}}}_{n\,}:=\left\{\left(\frac{{{\mathbb{1}}}^{\otimes n}+{X}_{n}}{2},\,\frac{{{\mathbb{1}}}^{\otimes n}-{X}_{n}}{2}\right):\,{X}_{n}={X}_{n}^{\dagger }\in {\mathcal{L}}({{\mathcal{H}}}^{\otimes n}),\,\parallel \!{X}_{n}{\parallel }_{\infty }\le {r}_{n}\right\},$$
  where ∥ ⋅ ∥_*∞*_ denotes the operator norm, is *compatible* with ${({{\mathcal{F}}}_{n})}_{n}$ (refs. ^42,63^). This means that whenever a measurement ${\mathcal{M}}\in {{\mathbb{M}}}_{n}$ is performed on the first *n* subsystems of a free state $\sigma \in {{\mathcal{F}}}_{n+m}$, the resulting post-measurement state on the last *m* subsystems is also a free state in ${{\mathcal{F}}}_{m}$ for each one of the two possible outcomes of ${\mathcal{M}}$. Here, $\mathcal{L}({\mathcal{H}}^{\otimes n})$ denotes the space of linear operators acting on the Hilbert space ${\mathcal{H}}^{\otimes n}$*.*

Aside from the fact that both are obeyed by the set of separable states, as we show in Supplementary Note D, it is a priori unclear why Axiom 6’ is in any way related to Axiom 6. The connection between the two follows from the work of Piani^63^, who proved that Axiom 6 is satisfied whenever one can find a tomographically complete set of measurements that is compatible with the free states; the sets ${{\mathbb{M}}}_{n}$ in equation (20) are, in fact, tomographically complete because the POVM operators $({{\mathbb{1}}}^{\otimes n}+{X}_{n})/2$ span the space of Hermitian operators on ${{\mathcal{H}}}^{\otimes n}$. It turns out that Axiom 6’ is what we need to prove the generalized quantum Sanov’s theorem.

In the following, our proof strategy will be to:

1. Derive the generalized Sanov’s theorem for the commutative case of sets of classical states ${{\mathcal{F}}}_{n}$ that respect Axioms 1–6 (sections ‘Max-relative entropy and the blurring lemma’ and ‘Classical generalized Sanov’s theorem’).
2. Choose suitable measurement operations for lifting the result to the non-commutative (quantum) setting, assuming Axioms 1–5 as well as Axiom 6’ (section ‘Lifting from classical to quantum’).

### Max-relative entropy and the blurring lemma

Instead of directly working with the hypothesis-testing relative entropy ${D}_{{\rm{H}}}^{\varepsilon }(\sigma \| \rho )$, our proofs start with a dual formulation in terms of the smooth max-relative entropy, which is defined as

21 $${D}_{\max }^{\varepsilon }(\sigma \| \rho ):={\log }_{2}\,\inf \Big\{\mu \in {\mathbb{R}}\,\Big| \,\widetilde{\sigma }\le \mu \rho ,\,\frac{1}{2}\| \widetilde{\sigma }-\sigma {\| }_{1}\le \varepsilon \Big\},$$

where we choose to measure the *ε*-closeness of states in terms of the trace distance. The smooth max-relative entropy enjoys, for any *δ* > 0 small enough, the duality relation^64–66^

22 $${D}_{\max }^{\sqrt{1-\varepsilon }}(\sigma \| \rho )\le {D}_{{\rm{H}}}^{\varepsilon }(\sigma \| \rho )\le {D}_{\max }^{1-\varepsilon -\delta }(\sigma \| \rho )+{\log }_{2}\frac{1}{\delta },$$

which implies that we can essentially replace the hypothesis-testing relative entropy with the smooth max-relative entropy, up to suitably modifying the smoothing parameter.

The generalized Sanov’s theorem for general sets of states ${\mathcal{F}}$ then becomes equivalent to

23 $$\displaystyle \mathop{\mathrm{lim}}\limits_{n\to \infty }\frac{1}{n}\,{D}_{\max }^{\varepsilon }({{\mathcal{F}}}_{n}\,\| \,{\rho }^{\otimes n})\mathop{=}\limits^{?}D({\mathcal{F}}\| \rho )\quad\forall \,\varepsilon \in (0,1),$$

and, using standard entropic arguments, it is not too difficult to show the special case *ε* → 0. Further, because the function on the left-hand side of equation (23) is monotonically non-increasing in *ε*, we immediately have that ${\mathrm{lim}}_{n\to \infty }\frac{1}{n}\,{D}_{\max }^{\varepsilon }({{\mathcal{F}}}_{n}\,\| \,{\rho }^{\otimes n})\le D({\mathcal{F}}\| \rho )$; consequently, it remains to prove the opposite direction. By contradiction, our goal will be to show for the classical case that

24 $$\frac{1}{n}\,{D}_{\max }^{\varepsilon }({{\mathcal{F}}}_{n}\,\| \,{p}^{\otimes n})\mathop{\longrightarrow }\limits_{n\to \infty }\lambda < D({\mathcal{F}}\| p),$$

under the assumption of Axioms 1–6.

The crucial tool for working with classical non-i.i.d. distributions in ${{\mathcal{F}}}_{n}$ is the blurring lemma recently established by one of us^11^. Namely, for any pair of positive integers $n,m\in {{\mathbb{N}}}_{+}$, one defines the blurring map ${B}_{n,m}:{{\mathbb{R}}}^{{{\mathcal{X}}}^{n}}\to {{\mathbb{R}}}^{{{\mathcal{X}}}^{n}}$, which transforms any input probability distribution by adding *m* symbols of each kind $x\in {\mathcal{X}}$, where $X$ is a a finite alphabet, shuffling the resulting sequence and discarding *m* symbols.

To better understand the action of this map, it is useful to recall some concepts from the theory of types^67^. The type of a sequence *x*^*n*^ of *n* symbols from a finite alphabet ${\mathcal{X}}$, denoted ${t}_{{x}^{n}}$, is simply the empirical probability distribution of the symbols of ${\mathcal{X}}$ found in *x*^*n*^: in other words, ${t}_{{x}^{n}}(x)=\frac{1}{n}\,N(x| {x}^{n})$, where *N*(*x*∣*x*^*n*^) denotes the number of times *x* appears in the sequence *x*^*n*^. The set of *n*-types is denoted as ${{\mathcal{T}}}_{n}$ (we regard the alphabet as fixed). A standard counting argument reveals that the number of types is only polynomial in *n*, unlike the number of possible sequences *x*^*n*^, which is exponential. More precisely, we have the estimate $| {{\mathcal{T}}}_{n}| \le {(n+1)}^{| {\mathcal{X}}| -1}$. This means that the size of the type classes, which comprise the set of sequences of a given type, is generically exponential. In what follows, we will indicate with *T*_*n*,*t*_ the type class associated with a given *n*-type $t\in {{\mathcal{T}}}_{n}$. Clearly, the union of all the type classes reproduces the set of all sequences.

An important observation for us is that any probability distribution *p*_*n*_ on ${{\mathcal{X}}}^{n}$ that is invariant under permutations, which means that the probability of two sequences that differ only by the order of the symbols is the same, can be understood in the space of types rather than in the space of sequences. In other words, such a probability distribution is uniquely specified by the values *p*_*n*_(*T*_*n*,*t*_) that it assigns to each type class. The essence of the blurring lemma, as stated below in equation (25), is the analysis of the effect that the above blurring map has in type space. As blurring perturbs the type of the input sequence a little in a random way, this action amounts to an effective ‘smearing’ of the input probability distribution in type space: a little of the probability weight that every type class carries ‘spills over’ to neighbouring type classes.

More quantitatively, the classical one-shot blurring lemma from ref. ^11^ (Lemma 9) then tells us that for *δ*, *η* > 0 and ${p}_{n},{q}_{n}\in {\mathcal{P}}({{\mathcal{X}}}^{n})$ permutationally symmetric with ${p}_{n}\big({\bigcup }_{t\in {{\mathcal{T}}}_{n}:\parallel s-t{\parallel }_{\infty }\le \delta }{T}_{n,t}\big)\ge 1-\eta$, we have

25 $${D}_{\max}^{\eta }({p}_{n}\,\| \,{B}_{n,m}({q}_{n}))\le {\log }_{2}\frac{1}{{q}_{n}({\cup }_{t\in {T}_{n}:\parallel s-t{\parallel }_{\infty }\le \delta }{T}_{n,t})}+ng\left(\left(2\delta +\frac{1}{n}\right)|X|\right),$$

for *m* = ⌈2*δn*⌉ and with the fudge function $$g(x)\,:=(x+1)\,{\log }_{2}(x+1)$$$$-x\,{\log }_{2}x$$. Refer to Supplementary Note C for more details and to Lemma 9 in ref. ^11^ for a detailed technical derivation.

### Classical generalized Sanov’s theorem

We will now attempt to give an intuitive but mathematically non-rigorous description of the proof of the classical version of Sanov’s theorem, which states that ${\rm{S}}{\rm{a}}{\rm{n}}{\rm{o}}{\rm{v}}(p\|{\mathcal{F}})=D({\mathcal{F}}\| p)$ under Axioms 1–6 in section ‘Axiomatic approach’. Following section ‘Max-relative entropy and the blurring lemma’ and, in particular, equation (24), by contradiction we can then construct two sequences of *ε*-close probability distributions ${q}_{n}^{{\prime} },{q}_{n}$, with ${q}_{n}\in {{\mathcal{F}}}_{n}$, such that ${q}_{n}^{{\prime} }\le {2}^{n\lambda }{p}^{\otimes n}$.

To make sense of this inequality, we have to evaluate it on a cleverly chosen set. The key tool for doing that is a simple lemma by Sanov, sometimes also known, alas, as Sanov’s theorem. This tells us that^68^

26 $${p}^{\otimes n}(\{{x}^{n}:\,{t}_{{x}^{n}}\in {\mathcal{A}}\})\le {\rm{p}}{\rm{o}}{\rm{l}}{\rm{y}}(n)\,{2}^{-nD({\mathcal{A}}\parallel p)}$$

for any set of probability distributions ${\mathcal{A}}$. It is clear what to do now: by choosing ${\mathcal{A}}={{\mathcal{F}}}_{1}$, we get on the right-hand side the exponential factor ${2}^{n(\lambda -D({\mathcal{F}}\| p))}$, which goes to zero sufficiently fast to overcome the polynomial. Thus, we have that ${q}_{n}^{{\prime} }(\{{x}^{n}:\,{t}_{{x}^{n}}\in {{\mathcal{F}}}_{1}\})\mathop{\to }\limits_{n\to \infty }0$; in other words, a sequence drawn according to ${q}_{n}^{{\prime} }$ has asymptotically vanishing probability of having a free type, that is, a type in ${{\mathcal{F}}}_{1}$.

This, at first sight, may seem good, but it should make us immediately suspicious, because ${q}_{n}^{{\prime} }$ is supposed to be *ε*-close to a free probability distribution ${q}_{n}\in {{\mathcal{F}}}_{n}$. It thus holds that ${q}_{n}\big(\{{x}^{n}:\,{t}_{{x}^{n}}\notin {{\mathcal{F}}}_{1}\}\big)\gtrsim 1-\varepsilon$ asymptotically. That is, sequences drawn with respect to the free probability distribution *q*_*n*_ have a non-free type with an asymptotically non-vanishing probability.

Let us elaborate on this intuition. As there are only a polynomial number of types, the above reasoning shows that there exists a non-free type $s\notin {{\mathcal{F}}}_{1}$ such that ${q}_{n}({T}_{n,s})\gtrsim \frac{1-\varepsilon }{\mathrm{poly}(n)} \vphantom{\Big|}$. Of course, *s* might depend on *n*, but for now the reader will have to trust us that up to extracting converging subsequences, we can circumvent this obstacle (Supplementary Note C). So, now we have a free probability distribution *q*_*n*_ that has a substantial weight (only polynomially vanishing) on a certain type class *T*_*n*,*s*_ corresponding to a non-free type $s\notin {{\mathcal{F}}}_{1}$.

Enter blurring. By blurring *q*_*n*_, we can make it have substantial weight not only on *T*_*n*,*s*_ but on all type classes *T*_*n*,*t*_ with *t* ≈ *s*. This is what blurring does: it spreads weight around among close type classes. Hence, we will have that ${\widetilde{q}}_{n}({T}_{n,t})\gtrsim \frac{1-\varepsilon }{{\rm{p}}{\rm{o}}{\rm{l}}{\rm{y}}(n)\,{2}^{\alpha n}}$ for all *t* ≈ *s*, where *α* > 0 is a very small exponential price we have to pay to blur *q*_*n*_ into ${\widetilde{q}}_{n}$. For a more quantitative understanding of this phenomenon, we refer the reader to equation (25) and to the full technical proof in Supplementary Note C.

Now, because ${\widetilde{q}}_{n}$ has substantial weight in a whole neighbourhood of types around *s*, it becomes ideally suited to dominate probability distributions that are very concentrated there. There is an obvious candidate for one such distribution, and it is *s*^⊗*n*^ itself! What this reasoning will eventually show is that

27 $${s}^{\otimes n}\lesssim \frac{{\rm{p}}{\rm{o}}{\rm{l}}{\rm{y}}(n)\,{2}^{\alpha n}}{1-\varepsilon }\,{\widetilde{q}}_{n},$$

where in ≲ we have swept under the carpet the fact that *s*^⊗*n*^ needs to be deprived of its exponentially vanishing non-typical tails for this entry-wise inequality to work.

Now we are basically done. Because blurring does not increase the max-relative entropy of a resource significantly, it is possible to find a free probability distribution ${r}_{n}\in {{\mathcal{F}}}_{n}$ such that ${\widetilde{q}}_{n}\le {2}^{\beta n}{r}_{n}$ for some small *β* > 0. Chaining the inequalities will give us

28 $${s}^{\otimes n}\lesssim \frac{\mathrm{poly}(n)\,{2}^{(\alpha +\beta )n}}{1-\varepsilon }\,{r}_{n}\,,$$

which, by the asymptotic equipartition property expressed as^7,69^

29 $$\mathop{\mathrm{lim}}\limits_{\varepsilon \to 0}\,\mathop{{\mathrm{lim}}\,{\mathrm{inf}}}\limits_{n\to \infty }\frac{1}{n}\,{D}_{\max }^{\varepsilon }({s}^{\otimes n}\|{\mathcal{F}}_{n})=D^{\infty }(s\| {\mathcal{F}}),$$

eventually implies that ${D}^{\infty }(s\| {\mathcal{F}})=0$. This is in direct contradiction with Axiom 6, and this contradiction will complete the proof.

A full technical proof following the argument sketched above is given in Supplementary Note C.

As a by-product of our argument, it is actually possible to design a simple explicit test that is asymptotically nearly optimal for the hypothesis task at hand. Namely, given a string of symbols ${x}^{n}\in {{\mathcal{X}}}^{n}$ and some small tolerance *ζ* > 0:

- If $\frac{1}{2}{\parallel {t}_{{x}^{n}}-{{\mathcal{F}}}_{1}\parallel }_{1}\le \zeta$, where ${t}_{{x}^{n}}$ is the type of *x*^*n*^, then we guess that the underlying probability distribution is free.
- Otherwise, we guess that it is *p*.

This test can be shown to achieve an asymptotically vanishing type II error probability in the limit when *n* → *∞* and a type I error exponent that is approximately equal to the reverse relative entropy $D({\mathcal{F}}\| p)$, if *ζ* > 0 is sufficiently small.

### Lifting from classical to quantum

Once a solution of the classical problem has been established, we need to extend it to quantum systems. To do this, a standard strategy is to measure: indeed, quantum measurements map quantum states to classical probability distributions, so we can use them to bring the problem to a form that we can tackle with our classical result.

In the context of hypothesis testing, and, more specifically, resource testing—where, remember, we have to distinguish between a state *ρ*^⊗*n*^ and a generic free state ${\sigma }_{n}\in {{\mathcal{F}}}_{n}$—a possible strategy could be the following: we could choose a suitable measurement ${\mathcal{M}}$ with outcomes labelled by $x\in {\mathcal{X}}$, with ${\mathcal{X}}$ some finite alphabet, and carry it out on every copy of the system we have been given. By doing so, we map the problem into a classical resource-testing problem in which we have to distinguish between *p*^⊗*n*^, with $p:={\mathcal{M}}(\rho )$ being the probability distribution obtained by measuring *ρ*, and a generic free distribution ${q}_{n}:={{\mathcal{M}}}^{\otimes n}({\sigma }_{n})$, with ${\sigma }_{n}\in {{\mathcal{F}}}_{n}$.

Calling ${\widetilde{\mathcal{F}}}_n$ the set of *q*_*n*_’s obtained in this way, we can now try to apply the classical version of our generalized Sanov’s theorem to this set. To do this, one simply needs to verify Axioms 1–6 in section ‘Axiomatic approach’ for this sequence of sets $({\widetilde{\mathcal{F}}}_n)_n$. Although Axioms 1–5 are relatively straightforwardly checked, verifying Axiom 6 requires a more technically complex attack. We solve this problem by showing that Axiom 6’ at the quantum level directly implies Axiom 6 for the classical sets ${\widetilde{\mathcal{F}}}_n$ (see Theorem 14 in Supplementary Note D for details). Entanglement theory also satisfies Axiom 6’ (as proven in Corollary 15), so we can proceed. Applying our classical generalized Sanov’s theorem, we know that this strategy yields a type I error decay equal to

30 $${\mathrm{Sanov}}(\rho \| {\mathcal{F}})\ge \mathop{\min }\limits_{q\in {\widetilde{\mathcal{F}}}_{1}}D(q\| p)=\mathop{\min }\limits_{\sigma \in {{\mathcal{F}}}_{1}}D({\mathcal{M}}(\sigma )\| {\mathcal{M}}(\rho )).$$

Note that the first inequality holds because what we describe is a physically possible strategy, so it yields a lower bound on the Sanov exponent. We can now further optimize over the measurement ${\mathcal{M}}$, which yields the bound

31 $${\rm{S}}{\rm{a}}{\rm{n}}{\rm{o}}{\rm{v}}(\rho \| {\mathcal{F}})\ge \mathop{\min }\limits_{\sigma \in {{\mathcal{F}}}_{1}}{D}^{{\mathbb{ALL}}}(\sigma \| \rho )\,.$$

Here ${D}^{{\mathbb{ALL}}}(\sigma \| \rho )$ is the measured relative entropy^70^ between *σ* and *ρ*, optimized over all possible measurements.

However, we are not done yet, because the above expression is, in general, not equal to $\mathop{\min }\limits_{\sigma \in {{\mathcal{F}}}_{1}}D(\sigma \| \rho )=D({\mathcal{F}}\| \rho )$ due to the action of the measurement, which, in general, decreases the relative entropy distance between states^71^. To fix this remaining issue, we adopt a double-blocking procedure. In practice, before measuring, we group the *n* systems we have at our disposal into groups of *k* systems each (discarding the rest, if any); here *k* is a fixed constant. By doing so we obtain that

32 $${\rm{S}}{\rm{a}}{\rm{n}}{\rm{o}}{\rm{v}}(\rho \| {\mathcal{F}})\ge \mathop{\min }\limits_{\sigma \in {{\mathcal{F}}}_{1}}\frac{1}{k}\,{D}^{{\mathbb{ALL}}}\big({\sigma }_{k}\,\| \,{\rho }^{\otimes k}\big).$$

Optimizing over *k* gives the main claim, because, by the entropic pinching inequality (Lemma 4.11 in ref. ^72^), the right-hand side converges to $D({\mathcal{F}}\| \rho )$ as *k* → *∞*, as claimed. Like the classical case, it is also possible in the quantum case to describe a nearly optimal test (a measurement) for resource testing, although in a less explicit way due to the lifting procedure involved.

Full details of the proof are given in Supplementary Note D.

## Online content

Any methods, additional references, Nature Portfolio reporting summaries, source data, extended data, supplementary information, acknowledgements, peer review information; details of author contributions and competing interests; and statements of data and code availability are available at 10.1038/s41567-026-03182-x.

## Supplementary information

Supplementary Information Supplementary Notes A–E, which contain technical details and proofs.

## Data Availability

No datasets were generated or analysed during this study.
