# Supplementary material for: Asymptotic quantification of entanglement with a single copy
Source: Nat Phys. 2026 Mar 13;22(3):439–45. doi: 10.1038/s41567-026-03182-x (PMC13046469; doi:10.1038/s41567-026-03182-x)
Supplement: Supplementary file 1 — Supplementary Notes A–E, which contain technical details and proofs. [file 41567_2026_3182_MOESM1_ESM.pdf]

nature physics

Supplementary information https://doi.org/10.1038/s41567-026-03182-x

Asymptotic quantification of entanglement with a single copy

In the format provided by the authors and unedited

CONTENTS

A. Proof of Lemma 1 1

B. Sanov's theorem: notation and definitions 4
1. Quantum setting with Brandão–Plenio axioms 4
2. Classical setting and the method of types 4
3. Relative entropies 5
4. Filtered divergences and compatibility conditions 5

C. Generalised classical Sanov's theorem 7
1. A key tool: the blurring lemma 8
2. Proof of the generalised Sanov's theorem: easy part 9
3. Proof of the generalised Sanov's theorem: hard part 10
4. On the optimal test in the classical case 13
5. How to verify Axiom 6 14

D. Generalised quantum Sanov's theorem 14

E. Further considerations 20
1. Additivity violation for the reverse Rényi relative entropy of entanglement 20
2. A (classical) counter-example 21

References 23

Appendix A: Proof of Lemma 1

We recall the definitions of the two relevant quantities. On the one hand, the hypothesis testing relative entropy of entanglement testing is

$$\begin{aligned} D_H^\varepsilon(\mathcal{S}_{A:B}\|\rho_{AB}) &= \min_{\sigma_{AB}\in\mathcal{S}_{A:B}} D_H^\varepsilon(\sigma_{AB}\|\rho_{AB}) \\ &= -\log_2 \max_{\sigma_{AB}\in\mathcal{S}_{A:B}} \min\left\{\operatorname{Tr} M\rho_{AB}\ \middle|\ 0\le M\le \mathbb{1},\ \operatorname{Tr} M\sigma \ge 1-\varepsilon\right\} \\ &= -\log_2 \min\left\{\operatorname{Tr} M\rho_{AB}\ \middle|\ 0\le M\le \mathbb{1},\ \operatorname{Tr} M\sigma \ge 1-\varepsilon\ \forall \sigma\in\mathcal{S}_{A:B}\right\}, \end{aligned} \tag{A1}$$

where the equality between the second and third line follows from von Neumann's minimax theorem [1]. The Sanov exponent is then given by

$$\mathrm{Sanov}(\rho_{AB}\|\mathcal{S}_{A:B}) = \lim_{\varepsilon\to 0}\liminf_{n\to\infty}\frac{1}{n}D_H^\varepsilon(\mathcal{S}_{A^n:B^n}\|\rho_{AB}^{\otimes n}). \tag{A2}$$

On the other hand, the distillation exponent for m copies of the maximally entangled state

$|\Phi_+\rangle$ is

$$E_{d,\text{err}}^{(m)}(\rho_{AB}) = \sup\left\{ \liminf_{n\to\infty} -\frac{1}{n}\log_2 \varepsilon_n \;\middle|\; F\big(\Lambda_n(\rho_{AB}^{\otimes n}),\, |\Phi_m\rangle\langle\Phi_m|\,\big) \geq 1-\varepsilon_n,\ \Lambda_n \in \text{NE} \right\}, \tag{A3}$$

where $|\Phi_m\rangle := |\Phi_+\rangle^{\otimes m}$ and $F(\rho,\sigma) = \left\|\sqrt{\rho}\sqrt{\sigma}\right\|_1^2$ denotes the fidelity. The asymptotic error exponent of entanglement distillation is then

$$E_{d,\text{err}}(\rho_{AB}) = \lim_{m\to\infty} E_{d,\text{err}}^{(m)}(\rho_{AB}). \tag{A4}$$

At this point, two remarks are in order. First, we should comment about the terminology 'error exponent'. Error exponents are typically defined slightly differently in traditional Shannon information theory (see [2] for a quantum example): namely, in common tasks one often looks for the optimal exponent of error decay under a fixed rate $r := \frac{n}{m(n)}$, whereas we do not fix the precise number m of maximally entangled copies to obtain, only requiring that $m \to \infty$ as $n \to \infty$.

Second, one could alternatively employ the trace distance $\frac{1}{2}\left\|\Lambda_n(\rho_{AB}^{\otimes n}) - |\Phi_m\rangle\langle\Phi_m|\right\|_1$ as an error metric in Eq. (A3). The choice between the (in)fidelity and the trace distance is immaterial in the task of distillation, and all results will be exactly the same.

Lemma 1. *The asymptotic error exponent of entanglement distillation under non-entangling operations equals the Sanov error exponent of hypothesis testing of all separable states $\mathcal{S}_{A:B}$ against ρ_{AB}:*

$$E_{d,\text{err}}(\rho_{AB}) = \text{Sanov}(\rho_{AB}\|\mathcal{S}_{A:B}). \tag{A5}$$

Proof. Consider any feasible distillation protocol $\Lambda_n \in \text{NE}$ such that

$$\begin{aligned} 1-\varepsilon_n &\leq F\big(\Lambda_n(\rho_{AB}^{\otimes n}),\, |\Phi_m\rangle\langle\Phi_m|\,\big) \\ &= \langle\Phi_m|\,\Lambda_n(\rho_{AB}^{\otimes n})\,|\Phi_m\rangle \\ &= \operatorname{Tr}\left[\rho_{AB}^{\otimes n}\,\Lambda_n^\dagger(|\Phi_m\rangle\langle\Phi_m|)\right] \end{aligned} \tag{A6}$$

where $\Lambda_n^\dagger$ denotes the adjoint map of Λ_n. Define $M_n = \mathbb{1} - \Lambda_n^\dagger(|\Phi_m\rangle\langle\Phi_m|)$, which is a valid POVM element since Λ_n is a quantum channel. Notice now that, for any $\sigma_n \in \mathcal{S}_{A^n:B^n}$, it holds that

$$\begin{aligned} \operatorname{Tr} M_n\sigma_n &= 1 - \langle\Phi_m|\Lambda_n(\sigma_n)|\Phi_m\rangle \\ &\geq 1 - \max_{\sigma_n' \in \mathcal{S}_{A^n:B^n}} \langle\Phi_m|\sigma_n'|\Phi_m\rangle \\ &\geq 1 - 2^{-m}, \end{aligned} \tag{A7}$$

where the second line follows since Λ_n is non-entangling, and the last line is a basic property of the maximally entangled state [3]. Entanglement testing with the measurement $(M_n, \mathbb{1} - M_n)$ thus gives a general upper bound on the distillation error exponent as

$$\min_{\sigma_n\in\mathcal{S}_{A^n:B^n}} D_H^{2^{-m}}(\sigma_n\|\rho_{AB}^{\otimes n}) \geq \log_2 \frac{1}{\operatorname{Tr} M_n \rho_{AB}^{\otimes n}} \geq -\log_2 \varepsilon_n. \tag{A8}$$

Dividing by n, taking the supremum over $\Lambda_n \in \text{NE}$, and then the limit $n \to \infty$, yields

$$E_{d,\text{err}}^{(m)}(\rho_{AB}) \leq \liminf_{n\to\infty} \frac{1}{n} \min_{\sigma_n\in\mathcal{S}_{A^n:B^n}} D_H^{2^{-m}}(\sigma_n\|\rho_{AB}^{\otimes n}) = \liminf_{n\to\infty} \frac{1}{n} D_H^{2^{-m}}(\mathcal{S}_{A^n:B^n}\|\rho_{AB}^{\otimes n}). \tag{A9}$$

For the other direction, consider any measurement operator M_n satisfying $\operatorname{Tr} M_n \sigma \ge 1 - 2^{-m}$ for all separable σ. Construct the map Λ_n acting on the system $A^n B^n$, producing at the output an $(m+m)$-qubit operator, and defined as

$$\Lambda_n(\cdot) = \operatorname{Tr}[(\mathbb{1} - M_n)(\cdot)]\, |\Phi_m\rangle\langle\Phi_m| + \operatorname{Tr}[M_n(\cdot)]\, \frac{\mathbb{1} - |\Phi_m\rangle\langle\Phi_m|}{2^{2m}-1}\,. \tag{A10}$$

We immediately have that $F\big(\Lambda_n(\rho_{AB}^{\otimes n}), |\Phi_m\rangle\langle\Phi_m|\big) = \operatorname{Tr}\big[(\mathbb{1} - M_n)\rho^{\otimes n}\big]$. Furthermore, for any $\sigma_n \in \mathcal{S}_{A^n:B^n}$ it holds that

$$\begin{aligned}\Lambda_n(\sigma_n) &= \operatorname{Tr}[(\mathbb{1} - M_n)\sigma_n]\left(|\Phi_m\rangle\langle\Phi_m| + \frac{\operatorname{Tr} M_n\sigma_n}{1 - \operatorname{Tr} M_n \sigma_n}\,\frac{\mathbb{1} - |\Phi_m\rangle\langle\Phi_m|}{2^{2m}-1}\right)\\ &= \operatorname{Tr}[(\mathbb{1} - M_n)\sigma_n]\left(|\Phi_m\rangle\langle\Phi_m| + (2^m - 1)\,\frac{\mathbb{1} - |\Phi_m\rangle\langle\Phi_m|}{2^{2m}-1} + \eta\,\frac{\mathbb{1} - |\Phi_m\rangle\langle\Phi_m|}{2^{2m}-1}\right)\end{aligned} \tag{A11}$$

for some $\eta \ge 0$, since $\operatorname{Tr} M_n\sigma_n \ge 1 - 2^{-m}$ by assumption. But since both $\frac{\mathbb{1} - |\Phi_m\rangle\langle\Phi_m|}{2^{2m}-1}$ and $|\Phi_m\rangle\langle\Phi_m| + (2^m-1)\,\frac{\mathbb{1} - |\Phi_m\rangle\langle\Phi_m|}{2^{2m}-1}$ are separable states [4], this means that $\Lambda_n(\sigma_n)$ is also separable, and hence Λ_n is a non-entangling map. Thus

$$E_{d,\mathrm{err}}^{(m)}(\rho_{AB}) \ge \liminf_{n\to\infty} \frac{1}{n} \log_2 \frac{1}{\operatorname{Tr} M_n \rho^{\otimes n}}, \tag{A12}$$

and optimising over all feasible measurements gives

$$E_{d,\mathrm{err}}^{(m)}(\rho_{AB}) \ge \liminf_{n\to\infty} \frac{1}{n}\, D_H^{2^{-m}}(\mathcal{S}_{A^n:B^n} \| \rho_{AB}^{\otimes n})\,. \tag{A13}$$

Finally, noting that the function D_H^{ε} is monotonically non-decreasing in ε, we have that

$$\lim_{\varepsilon\to 0} \liminf_{n\to\infty} \frac{1}{n}\, D_H^{\varepsilon}(\mathcal{S}_{A^n:B^n} \| \rho_{AB}^{\otimes n}) = \lim_{m\to\infty} \liminf_{n\to\infty} \frac{1}{n}\, D_H^{2^{-m}}(\mathcal{S}_{A^n:B^n} \| \rho_{AB}^{\otimes n}) \tag{A14}$$

since the limit $\varepsilon \to 0$ exists and can be taken along any sequence, in particular $\varepsilon = 2^{-m}$ with $m \to \infty$. Combining (A9), (A13), and (A14), we thus infer that

$$E_{d,\mathrm{err}}(\rho_{AB}) = \lim_{m\to\infty} E_{d,\mathrm{err}}^{(m)}(\rho) = \lim_{m\to\infty} \liminf_{n\to\infty} \frac{1}{n}\, D_H^{2^{-m}}(\mathcal{S}_{A^n:B^n} \| \rho_{AB}^{\otimes n}) = \operatorname{Sanov}(\rho_{AB} \| \mathcal{S}_{A:B})\,, \tag{A15}$$

concluding the proof. ■

Remark. We will later see that the number of target states m is actually irrelevant for the result, and in fact it holds that

$$E_{d,\mathrm{err}}^{(m)}(\rho_{AB}) = E_{d,\mathrm{err}}(\rho_{AB}) = \lim_{n\to\infty} \frac{1}{n} D_H^{\varepsilon}(\mathcal{S}_{A:B} \| \rho_{AB}^{\otimes n}) = D(\mathcal{S}_{A:B} \| \rho) \tag{A16}$$

for all m and all $\varepsilon \in (0,1)$. This follows from the strong converse property of the generalised quantum Sanov's theorem (see Supplementary Note D).

Remark. The result of Lemma 1 holds not only in the case of separable states and non-entangling operations: the exact same proof applies to the case where the set $\mathcal{S}_{A:B}$ in Eq. (A2) is replaced with the set $\mathcal{PPT}_{A:B}$ of states with positive partial transpose (PPT), and analogously non-entangling operations NE in Eq. (A3) are replaced with PPT-preserving channels, i.e. ones that map PPT states to PPT states. This is essentially because states of the form encountered in Eq. (A11), known as isotropic states, are separable if and only if they are PPT [3]. This will in fact allow not only for Lemma 1 but also our main result in Theorem 2 to apply to the case where the free states are PPT states. (This immediate application of our methods to PPT states was also observed by Zhiwen Lin [5] after the initial preprint of this manuscript appeared online.)

Appendix B: Sanov's theorem: notation and definitions

1. Quantum setting with Brandão–Plenio axioms

Let $\mathcal{H}$ be a Hilbert space of finite dimension d. We consider a family $(\mathcal{F}_n)_n$ of sets of 'free states'. In practice, each $\mathcal{F}_n$ is a subset of the set of density operators on n copies of the system, in formula $\mathcal{F}_n \subseteq \mathcal{D}(\mathcal{H}^{\otimes n})$. The sets of free states should satisfy some elementary properties, called the *Brandão–Plenio axioms* [6, p. 795]:

1. Each $\mathcal{F}_n$ is a convex and closed subset of $\mathcal{D}(\mathcal{H}^{\otimes n})$, and hence also compact (since $\mathcal{H}$ is finite dimensional).
2. $\mathcal{F}_1$ contains some full-rank state $\mathcal{F}_1 \ni \sigma_0 > 0$.
3. The family $(\mathcal{F}_n)_n$ is closed under partial traces, i.e. if $\sigma \in \mathcal{F}_{n+1}$ then $\mathrm{Tr}_{n+1}\,\sigma \in \mathcal{F}_n$, where Tr_{n+1} denotes the partial trace over the last subsystem.
4. The family $(\mathcal{F}_n)_n$ is closed under tensor products, i.e. if $\sigma \in \mathcal{F}_n$ and $\sigma' \in \mathcal{F}_m$ then $\sigma \otimes \sigma' \in \mathcal{F}_{n+m}$.
5. Each $\mathcal{F}_n$ is closed under permutations, i.e. if $\sigma \in \mathcal{F}_n$ and $\pi \in S_n$ denotes an arbitrary permutation of a set of n elements, then also $U_\pi \sigma U_\pi^\dagger \in \mathcal{F}_n$, where U_π is the unitary implementing π over $\mathcal{H}^{\otimes n}$.

These axioms are conceived as an abstraction of the properties of the set of separable states. But there is one more subtle property of the set of separable states that is not captured by Axioms 1–5, and it is the fact that the regularised relative entropy of entanglement is faithful on entangled states, i.e. it is zero on a state if and only that state is separable. This highly non-trivial fact has been proved independently by Brandão and Plenio [6, Corollary II.2] and by Piani [7]. As shown in Supplementary Note E 2, such a conclusion does not follow solely from the above Axioms 1 – 5, so we will need to introduce an additional one:

6. The regularised relative entropy of resource is faithful, i.e.

$$\rho \notin \mathcal{F}_1 \quad \Longrightarrow \quad D^\infty(\rho \,\|\, \mathcal{F}) := \lim_{n\to\infty} \frac{1}{n} D(\rho^{\otimes n} \,\|\, \mathcal{F}_n) > 0\,. \tag{B1}$$

As usual, the existence of the limit on the right-hand side of (B1) is guaranteed by Axiom 4 (closure under tensor products) via Fekete's lemma [8].

2. Classical setting and the method of types

All of the above Axioms 1 – 6 make perfect sense for classical probability distributions, too. This is the case we are going to care about first, so we better fix some terminology. Let $\mathcal{X}$ be a finite alphabet of size $|\mathcal{X}|$. Denote with $\mathcal{P}(\mathcal{X})$ the set of probability distributions on $\mathcal{X}$. For a positive integer $n \in \mathbb{N}_+$, an ***n*-type** on $\mathcal{X}$ is a probability distribution $t \in \mathcal{P}(\mathcal{X})$ such that $nt(x) \in \mathbb{N}$ is an integer for all $n \in \mathbb{N}$. Therefore, the set of all n-types on $\mathcal{X}$ can be defined as

$$\mathscr{T}_n := \left\{ \left(\frac{k_j}{n}\right)_{j=1,\ldots,d} :\ k_j \in \mathbb{N}\ \ \forall\, j\,,\ \textstyle\sum_{j=1}^d k_j = n \right\}. \tag{B2}$$

In the above equation we did not indicate the dependency of $\mathcal{T}_n$ on $\mathcal{X}$, as we shall always assume that $\mathcal{X}$ is fixed. We will stick to this convention throughout the note in order to simplify the notation.

A well-known counting argument shows that

$$|\mathcal{T}_n| \leq \binom{n+d-1}{d-1} \leq (n+1)^{d-1}\,. \tag{B3}$$

For a given $t \in \mathcal{T}_n$, the associated ***type class*** $T_{n,t}$ is the set of sequences of length n made from elements in $\mathcal{X}$ that have type t. In formula,

$$T_{n,t} \coloneqq \{x^n \in \mathcal{X}^n :\ N(x|x^n) = nt(x)\ \ \forall\, x \in \mathcal{X}\}\,, \tag{B4}$$

where $N(x|x^n)$ denotes the number of times the symbol x appears in the sequence x^n. Clearly, any sequence in $T_{n,t}$ can be obtained from any other such sequence by applying a suitable permutation.

3. Relative entropies

For a generic divergence $\mathbb{D}$ and two sets of quantum states $\mathcal{A}, \mathcal{B} \subseteq \mathcal{D}(\mathcal{H})$, we write

$$\mathbb{D}(\mathcal{A}\|\mathcal{B}) \coloneqq \inf_{a\in\mathcal{A},\, b\in\mathcal{B}} D(a\|b)\,. \tag{B5}$$

In the case where $\mathcal{A} = \{a\}$ is composed of one element only, we use the shorthand notation $\mathbb{D}(a\|\mathcal{B})$, and similarly in the case $\mathcal{B} = \{b\}$. The same definitions can be given in the case where $\mathcal{A}, \mathcal{B} \subseteq \mathcal{P}(\mathcal{X})$ are sets of classical probability distributions.

4. Filtered divergences and compatibility conditions

Given a finite-dimensional quantum system A with Hilbert space $\mathcal{H}$, a ***measurement*** on A is a CPTP map $\mathcal{L}(\mathcal{H}) \to \mathbb{R}^N$, where $N \in \mathbb{N}^+$ is the number of outcomes (assumed to be finite). We can equivalently represent a measurement with N outcomes by an N-outcome POVM, i.e. a sequence $(E_x)_{x=1,\ldots,N}$ of positive semi-definite operators $E_x \geq 0$ such that $\sum_x E_x = \mathbb{1}$. Given a set $\mathbb{M}$ of measurements on A, one can use it to define a 'filtered' notion of relative entropy, by setting

$$D^{\mathbb{M}}(\rho\|\sigma) \coloneqq \sup_{\mathcal{M}\in\mathbb{M}} D\big(\mathcal{M}(\rho)\,\big\|\,\mathcal{M}(\sigma)\big)\,. \tag{B6}$$

A particularly simple choice of $\mathbb{M}$ is the set of *all* measurements on A, denoted by $\mathbb{ALL}$. It turns out that in this case one can relate the measured relative entropy, $D^{\mathbb{ALL}}(\rho\|\sigma)$, first considered by Donald [9] (see also [10, 11]), to the standard, Umegaki relative entropy, $D(\rho\|\sigma)$. This is done via the *pinching inequality* [12], which gives us the relation [13, Lemma 4.11] (see also [14, Eq. (47)])

$$D(\rho\|\sigma) - \log_2\big|\mathrm{spec}(\sigma)\big| \leq D^{\mathbb{ALL}}(\rho\|\sigma) \leq D(\rho\|\sigma)\,, \tag{B7}$$

where $|\,\mathrm{spec}(\sigma)|$ denotes the number of *distinct* eigenvalues of σ. Using the estimate [15, Eq. (8)]

$$\big|\mathrm{spec}\left(\sigma^{\otimes k}\right)\big| \leq \binom{k+d-1}{d-1} \leq \frac{(k+d-1)^{d-1}}{(d-1)!}\,, \tag{B8}$$

where d is the dimension of the underlying quantum system, one sees that for all states ρ_k on A^k and σ on A it holds that

$$D\left(\rho_k \,\middle\|\, \sigma^{\otimes k}\right) - \log_2 \frac{(k+d-1)^{d-1}}{(d-1)!} \leq D^{\mathbb{ALL}}\left(\rho_k \,\middle\|\, \sigma^{\otimes k}\right) \leq D\left(\rho_k \,\middle\|\, \sigma^{\otimes k}\right). \tag{B9}$$

The fact that the fudge term on the left-hand side of the above inequality is of the form $\log_2 \mathrm{poly}(k)$, and therefore dividing by k and taking the limit $k \to \infty$ makes it vanish, is at the heart of the *asymptotic spectral pinching* method [12, 15, 16].

For any two given sets $\mathcal{A}, \mathcal{B} \subseteq \mathcal{D}(\mathcal{H})$ and an arbitrary set of measurements $\mathbb{M}$, we can also plug the divergence $D^{\mathbb{M}}$ into (B5) and define a corresponding 'filtered distance' $D^{\mathbb{M}}(\mathcal{A}\|\mathcal{B})$, given by

$$D^{\mathbb{M}}(\mathcal{A}\|\mathcal{B}) = \inf_{\rho\in\mathcal{A},\,\sigma\in\mathcal{B}} D^{\mathbb{M}}(\rho\|\sigma) = \inf_{\rho\in\mathcal{A},\,\sigma\in\mathcal{B}} \sup_{\mathcal{M}\in\mathbb{M}} D\left(\mathcal{M}(\rho) \,\middle\|\, \mathcal{M}(\sigma)\right). \tag{B10}$$

There are at least two natural questions to ask at this point. First, under what conditions is $D^{\mathbb{M}}(\cdot\|\cdot)$ a faithful measure of statistical distance between quantum states? The key notion in this respect is that of *information completeness*.

Definition 4. *A set of measurements $\mathbb{M}$ on a quantum system with finite-dimensional Hilbert space $\mathcal{H}$ is said to be* **informationally complete** *if the statistics under all measurements in $\mathbb{M}$ suffice to reconstruct any state on $\mathcal{H}$. In mathematical terms, this is equivalent to*

$$\mathrm{span}\{E_x : (E_x)_x \in \mathbb{M}\} = \mathcal{L}(\mathcal{H}). \tag{B11}$$

If $\mathbb{M}$ is informationally complete, then it is not difficult to see that $D^{\mathbb{M}}(\cdot\|\cdot)$ is faithful over pairs of quantum states, i.e. $D^{\mathbb{M}}(\rho\|\sigma) > 0$ whenever $\rho \neq \sigma$.

Looking again at the definition of $D^{\mathbb{M}}(\mathcal{A}\|\mathcal{B})$, the second natural question to ask is whether the infimum and supremum on the right-hand side of (B10) can be exchanged. A sufficient condition that enables precisely that has been identified by Brandão, Harrow, Lee, and Peres in [17].

Definition 5 [17, Definition 5]**.** *A set of measurement $\mathbb{M}$ on a given quantum system is* **closed under finite labelled mixtures** *if for all finite alphabets $\mathcal{Y}$ and all collections of POVMs $\left(E_{x|y}\right)_x \in \mathbb{M}$ labelled by $y \in \mathcal{Y}$ and all probability distributions p on $\mathcal{Y}$ the POVM constructed by drawing a symbol in $\mathcal{Y}$ and performing the corresponding measurement $\left(E_{x|y}\right)_x$ is still in $\mathbb{M}$. In formula,*

$$\left(p(y)\, E_{x|y}\right)_{x,y} \in \mathbb{M}. \tag{B12}$$

The following lemma from [17] answers the above question.

Lemma 6. *Let $\mathcal{A}, \mathcal{B} \subseteq \mathcal{D}(\mathcal{H})$ be closed and convex sets of (finite-dimensional) quantum states, and let $\mathbb{M}$ be a set of quantum measurements on the same system. If $\mathbb{M}$ is closed under finite labelled mixtures according to Definition 5, then*

$$D^{\mathbb{M}}(\mathcal{A}\|\mathcal{B}) = \inf_{\rho\in\mathcal{A},\,\sigma\in\mathcal{B}} \sup_{\mathcal{M}\in\mathbb{M}} D\left(\mathcal{M}(\rho) \,\middle\|\, \mathcal{M}(\sigma)\right) = \sup_{\mathcal{M}\in\mathbb{M}} \inf_{\rho\in\mathcal{A},\,\sigma\in\mathcal{B}} D\left(\mathcal{M}(\rho) \,\middle\|\, \mathcal{M}(\sigma)\right), \tag{B13}$$

and both infima are achieved, i.e. they can be replaced by minima.

In what follows, we will consider sequences $(\mathbb{M}_n)_n$ of sets $\mathbb{M}_n$ of measurements on n copies of a given quantum system. When sets of restricted measurements are present alongside sets of restricted states, a key notion is that of compatibility between these two objects. We borrow the following definition from [17].

Definition 7 [17, Definition 4]. *Let $\mathcal{H}$ be a Hilbert space, $(\mathcal{F}_n)_n$ a sequence of sets of quantum states $\mathcal{F}_n \subseteq \mathcal{D}(\mathcal{H}^{\otimes n})$, and $(\mathbb{M}_n)_n$ a sequence of sets $\mathbb{M}_n$ of n-copy measurements on the corresponding quantum system. We say that $(\mathbb{M}_n)_n$ is* **compatible** *with $(\mathcal{F}_n)_n$ if for all $n, m \in \mathbb{N}^+$, for all POVM operators $E_x^{(n)}$ that appear in a measurement belonging to $\mathbb{M}_n$, and for all states $\rho_{n+m} \in \mathcal{F}_{n+m}$, it holds that*

$$\mathrm{Tr}_{1\ldots n}\left[\left(E_x^{(n)} \otimes \mathbb{1}^{\otimes m}\right) \rho_{n+m}\right] \in \mathrm{cone}(\mathcal{F}_m)\,, \tag{B14}$$

where $\mathrm{cone}(\mathcal{F}_m) := \{\lambda\sigma :\ \lambda \geq 0,\ \sigma \in \mathcal{F}_m\}$.

While the above definitions have all been given in the quantum setting, they can equally well be considered in the fully classical case. A *measurement* or *statistical test* on a classical system with (finite) alphabet $\mathcal{X}$ is simply a classical channel Λ with input $\mathcal{X}$ and output in another discrete alphabet $\mathcal{Y}$, represented by a conditional probability distribution $\Lambda(y|x)$. Information completeness of a set of *classical measurements* $\mathbb{L}$ simply means that

$$\mathrm{span}\left\{(\Lambda(y|x))_x :\ \Lambda \in \mathbb{L},\ \Lambda : \mathbb{R}^{\mathcal{X}} \to \mathbb{R}^{\mathcal{Y}},\ y \in \mathcal{Y}\right\} = \mathbb{R}^{\mathcal{X}}, \tag{B15}$$

where $(\Lambda(y|x))_x$ is thought of as a vector in $\mathbb{R}^{\mathcal{X}}$. Similarly, a sequence of sets of classical measurements $(\mathbb{L}_n)_n$ is compatible with a sequence of sets of classical probability distributions $(\mathcal{F}_n)_n$ if for all $n, m \in \mathbb{N}^+$, $q_{n+m} \in \mathcal{F}_{n+m}$, $\Lambda \in \mathbb{L}_n$ with $\Lambda : \mathbb{R}^{\mathcal{X}^n} \to \mathbb{R}^{\mathcal{Y}}$, and $y \in \mathcal{Y}$, it holds that $\tilde{q}_m \in \mathrm{cone}(\mathcal{F}_m)$, where

$$\tilde{q}_m(\tilde{x}^m) := \sum_{x^n} \Lambda(y|x^n)\, q_{n+m}(x^n \tilde{x}^m) \qquad \forall\, \tilde{x}^m \in \mathcal{X}^m. \tag{B16}$$

Whereas above supplementary material largely followed the literature, the methods presented in the following are completely novel.

Appendix C: Generalised classical Sanov's theorem

Given a sequence of sets of free states $\mathcal{F}_n$ and some $\rho \in \mathcal{D}(\mathcal{H})$, we consider the hypothesis testing task of distinguishing many i.i.d. copies of ρ from a generic free state $\sigma_n \in \mathcal{F}_n$. In contrast with the Stein setting, here we are interested in the rate of decay of the *type I* error. The corresponding exponent, called the ***Sanov exponent***, is formally defined as

$$\mathrm{Sanov}(\rho\|\mathcal{F}) := \lim_{\varepsilon\to 0^+} \liminf_{n\to\infty} \frac{1}{n} D_H^{\varepsilon}\left(\mathcal{F}_n \,\middle\|\, \rho^{\otimes n}\right), \tag{C1}$$

where as usual $D_H^{\varepsilon}\left(\mathcal{F}_n \,\middle\|\, \rho^{\otimes n}\right) = \inf_{\sigma_n\in\mathcal{F}_n} D_H^{\varepsilon}\left(\sigma_n \,\middle\|\, \rho^{\otimes n}\right)$.

The main result of this section consists in a single-letter expression for the above exponent in the classical case where $\rho = p \in \mathcal{P}(\mathcal{X})$ is a probability distribution. The Sanov exponent turns out to be equal to the ***reverse relative entropy of resource*** of p, given by

$$D(\mathcal{F}\|p) = \min_{q\in\mathcal{F}_1} D(q\|p)\,. \tag{C2}$$

Our main classical result is as follows.

Theorem 8 (Generalised classical Sanov's theorem)**.** *Let $\mathcal{X}$ be a finite alphabet, and let $(\mathcal{F}_n)_n$ be a sequence of sets of probability distributions $\mathcal{F}_n \subseteq \mathcal{P}(\mathcal{X}^n)$ that obeys Axioms 1–6 in Section B 1 (i.e. all the Brandão–Plenio axioms and in addition Axiom 6). Then it holds that*

$$\lim_{n\to\infty} \frac{1}{n} D_H^{\varepsilon}\big(\mathcal{F}_n \,\big\|\, p^{\otimes n}\big) = D(\mathcal{F}\|p) \qquad \forall\, p \in \mathcal{P}(\mathcal{X}), \quad \forall\, \varepsilon \in (0,1)\,, \tag{C3}$$

entailing in particular that

$$\mathrm{Sanov}(p\|\mathcal{F}) = D(\mathcal{F}\|p)\,. \tag{C4}$$

1. A key tool: the blurring lemma

For any pair of positive integers $n, m \in \mathbb{N}_+$ and, as usual, a fixed alphabet $\mathcal{X}$, we define the ***blurring map*** as a linear map $B_{n,m} : \mathbb{R}^{\mathcal{X}^n} \to \mathbb{R}^{\mathcal{X}^n}$ that transforms any input probability distribution by adding m symbols of each kind $x \in \mathcal{X}$, shuffling the resulting sequence, and discarding m symbols. In this way, if the input sequence is of length n, then the same is true of the output sequence. We can formalise the action of $B_{n,m}$ as

$$B_{n,m}(\cdot) := \mathrm{tr}_m\, \mathcal{S}_{n+m} \left((\cdot) \otimes \bigotimes\nolimits_x \delta_x^{\otimes m} \right), \tag{C5}$$

where δ_x denotes the deterministic probability distribution concentrated on x (i.e. such that $\delta_x(y) = 1$ if $y = x$, and $\delta_x(y) = 0$ otherwise). Note that the output of the blurring map is always permutationally symmetric. To quantify the effect of the blurring map we use the smooth max-relative entropy, whose definition for general quantum states we recall as [18, 19]

$$D^{\varepsilon}_{\max}(\sigma\|\rho) = \log_2 \inf \left\{ \mu \in \mathbb{R} \;\middle|\; \tilde{\sigma} \leq \mu\rho,\ \frac{1}{2}\|\tilde{\sigma} - \sigma\|_1 \leq \varepsilon \right\}. \tag{C6}$$

The following is a precise statement of (25) from the main text, taken from [20].

Lemma 9 (Classical one-shot blurring [20, Lemma 9])**.** *Let $p_n, q_n \in \mathcal{P}(\mathcal{X}^n)$ be two n-copy probability distributions, with p_n permutationally symmetric. For some $\delta, \eta > 0$, let p_n be $(1-\eta)$-concentrated on the δ-ball of n-types around a single-copy probability distribution $s \in \mathcal{P}(\mathcal{X})$, in the sense that*

$$p_n \left(\bigcup\nolimits_{t \in \mathcal{T}_n:\ \|s-t\|_\infty \leq \delta} T_{n,t} \right) \geq 1 - \eta\,, \tag{C7}$$

where $\|s-t\|_\infty := \max_{x\in\mathcal{X}} |s(x) - t(x)|$. Then for $m = \lceil 2\delta n \rceil$ it holds that

$$D^{\eta}_{\max}\big(p_n \,\big\|\, B_{n,m}(q_n)\big) \leq \log_2 \frac{1}{q_n \left(\bigcup_{t\in\mathcal{T}_n:\ \|s-t\|_\infty \leq \delta} T_{n,t} \right)} + n g\big(\big(2\delta + \tfrac{1}{n}\big) |\mathcal{X}| \big)\,, \tag{C8}$$

where the blurring map $B_{n,m}$ is defined by (C5), and the function g is the 'bosonic entropy function'

$$g(x) := (x+1)\log_2(x+1) - x\log_2 x\,. \tag{C9}$$

Note that if $q_n\big(\bigcup_{t\in\mathcal{T}_n:\ \|s-t\|_\infty\leq\delta} T_{n,t}\big) = 0$ then (C8) holds trivially with the convention that $\log_2 1/0 = \infty$.

2. Proof of the generalised Sanov's theorem: easy part

Throughout this subsection we will be analysing the fully quantum case. In the next one, instead, we will restrict ourselves to classical probability distributions.

Because of the weak/strong converse duality [21–23]

$$D_{\max}^{\sqrt{1-\varepsilon}}(\rho\|\sigma) \leq D_H^{\varepsilon}(\rho\|\sigma) \leq D_{\max}^{1-\varepsilon-\delta}(\rho\|\sigma) + \log_2 \frac{1}{\delta}, \tag{C10}$$

which holds for all $\varepsilon \in (0,1)$ and all $\delta \in (0, 1-\varepsilon]$, the generalised Sanov's theorem is equivalent to

$$\lim_{n\to\infty} \frac{1}{n} D_{\max}^{\varepsilon}\big(\mathcal{F}_n \,\big\|\, \rho^{\otimes n}\big) \overset{?}{=} D(\mathcal{F}\|\rho) \qquad \forall\, \rho \in \mathcal{D}(\mathcal{H}), \quad \forall\, \varepsilon \in (0,1)\,. \tag{C11}$$

We start by establishing that the above relation holds when $\varepsilon \to 0^+$. We first need a preliminary lemma.

Lemma 10. *Let $\mathcal{F} \subseteq \mathcal{D}(\mathcal{H})$ be a closed subset of states. For some $\rho \in \mathcal{D}(\mathcal{H})$ and $\varepsilon > 0$, define $D^{\varepsilon}(\mathcal{F}\|\rho) := \min_{\sigma':\, \frac12\|\sigma'-\mathcal{F}\|_1 \leq \varepsilon} D(\sigma'\|\rho)$, where $\|\sigma' - \mathcal{F}\|_1 := \min_{\sigma\in\mathcal{F}} \|\sigma' - \sigma\|_1$. Then it holds that*

$$\lim_{\varepsilon\to 0^+} D^{\varepsilon}(\mathcal{F}\|\rho) = D(\mathcal{F}\|\rho)\,. \tag{C12}$$

Proof. Clearly, $D^{\varepsilon}(\mathcal{F}\|\rho) \leq D(\mathcal{F}\|\rho)$ for all $\varepsilon > 0$. Therefore, we only have to prove that $\liminf_{\varepsilon\to0^+} D^{\varepsilon}(\mathcal{F}\|\rho) \geq D(\mathcal{F}\|\rho)$. Let $(\varepsilon_n)_n$ be a sequence of numbers $\varepsilon_n > 0$ such that $\lim_{n\to\infty}\varepsilon_n = 0$ and $\lim_{n\to\infty} D^{\varepsilon_n}(\mathcal{F}\|\rho) = \liminf_{\varepsilon\to0^+} D^{\varepsilon}(\mathcal{F}\|\rho)$. For all n, choose a point of minimum $\sigma'_n \in \mathcal{D}(\mathcal{H})$ of the lower semi-continuous function $D(\cdot\,\|\,\rho)$ on the closed set $\{\sigma' : \frac12\|\sigma' - \mathcal{F}\|_1 \leq \varepsilon_n\}$. Up to taking sub-sequences, we can assume that $\lim_{n\to\infty}\sigma'_n = \sigma$ converges. Note that since $\mathcal{F}$ is closed, it must be that $\sigma \in \mathcal{F}$. Now, using the lower semi-continuity of the relative entropy we have that

$$\liminf_{\varepsilon\to0^+} D^{\varepsilon}(\mathcal{F}\|\rho) = \lim_{n\to\infty} D^{\varepsilon_n}(\mathcal{F}\|\rho) = \lim_{n\to\infty} D(\sigma'_n\|\rho) \geq D(\sigma\|\rho) \geq D(\mathcal{F}\|\rho)\,, \tag{C13}$$

which concludes the proof. ■

Proposition 11. *Let $(\mathcal{F}_n)_n$ be a sequence of sets of states $\mathcal{F}_n \subseteq \mathcal{D}\big(\mathcal{H}^{\otimes n}\big)$ that obeys the Brandão–Plenio axioms (Axioms 1–5 in Section B 1). Then for all states $\rho \in \mathcal{D}(\mathcal{H})$ it holds that*

$$\lim_{\varepsilon\to0^+} \liminf_{n\to\infty} \frac{1}{n} D_{\max}^{\varepsilon}\big(\mathcal{F}_n \,\big\|\, \rho^{\otimes n}\big) = \lim_{\varepsilon\to0^+} \limsup_{n\to\infty} \frac{1}{n} D_{\max}^{\varepsilon}\big(\mathcal{F}_n \,\big\|\, \rho^{\otimes n}\big) = D(\mathcal{F}\|\rho)\,. \tag{C14}$$

Proof. Fix $\sigma \in \mathcal{F}_1$. Due to Axiom 4, we have that $\sigma^{\otimes n} \in \mathcal{F}_n$ for all $n \in \mathbb{N}^+$. Therefore

$$\begin{aligned} \lim_{\varepsilon\to0^+} \limsup_{n\to\infty} \frac{1}{n} D_{\max}^{\varepsilon}\big(\mathcal{F}_n \,\big\|\, \rho^{\otimes n}\big) &\leq \lim_{\varepsilon\to0^+} \limsup_{n\to\infty} \frac{1}{n} D_{\max}^{\varepsilon}\big(\sigma^{\otimes n} \,\big\|\, \rho^{\otimes n}\big) \\ &= D(\sigma\|\rho)\,, \end{aligned} \tag{C15}$$

where the last step is by the asymptotic equipartition property in the i.i.d. case [13, 24]. Minimising over $\sigma \in \mathcal{F}_1$, we obtain that

$$\lim_{\varepsilon\to0^+} \limsup_{n\to\infty} \frac{1}{n} D_{\max}^{\varepsilon}\big(\mathcal{F}_n \,\big\|\, \rho^{\otimes n}\big) \leq D(\mathcal{F}\|\rho)\,. \tag{C16}$$

For the other direction, we have that

$$
\begin{aligned}
D_{\max}^{\varepsilon}\left(\mathcal{F}_n \,\middle\|\, \rho^{\otimes n}\right) &\geq D^{\varepsilon}\left(\mathcal{F}_n \,\middle\|\, \rho^{\otimes n}\right) \\
&= \min_{\sigma'_n:\ \frac12\|\sigma'_n-\mathcal{F}_n\|_1\leq\varepsilon} D\left(\sigma'_n \,\middle\|\, \rho^{\otimes n}\right) \\
&\overset{\text{(i)}}{\geq} \min_{\sigma'_n:\ \frac12\|\sigma'_n-\mathcal{F}_n\|_1\leq\varepsilon} \sum_{j=1}^{n} D\left(\sigma'_{n,j} \,\middle\|\, \rho\right) \\
&\overset{\text{(ii)}}{\geq} n\, D^{\varepsilon}(\mathcal{F}\|\rho)\,.
\end{aligned}
\tag{C17}
$$

Here, in (i) we denoted with $\sigma'_{n,j}$ the reduced state of σ'_n on the jth subsystem. To justify (i), note that by the sub-additivity of entropy

$$
S(\sigma'_n) \leq \sum_{j=1}^{n} S\left(\sigma'_{n,j}\right), \tag{C18}
$$

which in turn implies that

$$
D\left(\sigma'_n \,\middle\|\, \rho^{\otimes n}\right) = -S(\sigma'_n) + \sum_{j=1}^{n}\left(S\left(\sigma'_{n,j}\right) + D\left(\sigma'_{n,j} \,\middle\|\, \rho\right)\right) \geq \sum_{j=1}^{n} D\left(\sigma'_{n,j} \,\middle\|\, \rho\right). \tag{C19}
$$

In (ii), instead, we simply observed that due to data processing and Axiom 3 it holds that

$$
\frac{1}{2}\left\|\sigma'_{n,j} - \mathcal{F}_1\right\|_1 \leq \frac{1}{2}\left\|\sigma'_n - \mathcal{F}_n\right\|_1 \leq \varepsilon \tag{C20}
$$

for all $j = 1, \ldots, n$. To conclude the proof, divide both sides of (C17) by n, take the limit inferior as $n \to \infty$ first and $\varepsilon \to 0^+$ second, and use Lemma 10. ■

3. Proof of the generalised Sanov's theorem: hard part

As anticipated, we now restrict ourselves to the classical case. At this point we know that

$$
\lim_{n\to\infty} \frac{1}{n} D_{\max}^{\varepsilon}\left(\mathcal{F}_n \,\middle\|\, p^{\otimes n}\right) \leq D(\mathcal{F}\|p) \qquad \forall\, p \in \mathcal{P}(\mathcal{X}), \quad \forall\, \varepsilon \in (0,1)\,, \tag{C21}
$$

simply because the function on the left-hand side is monotonically non-increasing in ε, and its limit for $\varepsilon \to 0^+$ corresponds to the right-hand side due to Proposition 11. To conclude, we need to prove the converse inequality. Before we start, let us recall a useful estimate due to Sanov himself, which, unfortunately, is sometimes also known as Sanov's theorem [25, 26]. See also the particularly clear formulation in [27, Exercise 2.12, p. 29].

Lemma 12. *Let $\mathcal{A} \subseteq \mathcal{P}(\mathcal{X})$ be a subset of probability distributions over a finite alphabet $\mathcal{X}$. Then for all $p \in \mathcal{P}(\mathcal{X})$ and for all $n \in \mathbb{N}^+$ it holds that*

$$
p^{\otimes n}\left(\{x^n:\ t_{x^n} \in \mathcal{A}\}\right) = p^{\otimes n}\left(\bigcup\nolimits_{t\in\mathcal{T}_n\cap\mathcal{A}} T_{n,t}\right) \leq (n+1)^{|\mathcal{X}|-1}\, 2^{-nD(\mathcal{A}\|p)}. \tag{C22}
$$

If $\mathcal{A}$ is convex, then the polynomial factor $(n+1)^{|\mathcal{X}|-1}$ in the rightmost side can be omitted.

We are now ready to provide the complete proof of the generalised classical Sanov's theorem (Theorem 8).

Proof of Theorem 8. As we just discussed, it suffices to show that

$$\liminf_{n\to\infty} \frac{1}{n} D_{\max}^{\varepsilon}\left(\mathcal{F}_n \,\middle\|\, p^{\otimes n}\right) \overset{?}{\geq} D(\mathcal{F}\|p) \qquad \forall\, p \in \mathcal{P}(\mathcal{X}), \quad \forall\, \varepsilon \in (0,1)\,. \tag{C23}$$

We will proceed by contradiction. Assume that for infinitely many values of n there exists

$$q_n' \in \mathcal{P}(\mathcal{X}^n)\,, \quad q_n \in \mathcal{F}_n\,, \quad \lambda < D(\mathcal{F}\|p)\,, \tag{C24}$$

such that

$$\frac{1}{2}\left\|q_n - q_n'\right\|_1 \leq \varepsilon\,, \quad q_n' \leq 2^{n\lambda} p^{\otimes n}, \tag{C25}$$

where in the second expression $\leq$ denotes entry-wise inequality between vectors in $\mathbb{R}^{\mathcal{X}^n}$. For the rest of this proof, unless otherwise specified, every limit $n \longrightarrow \infty$ is to be intended as taken on the diverging sequence of values of n for which (C24) – (C25) hold.

Due to Lemma 10, we can find some $\zeta > 0$ with the property that

$$D^{\zeta}(\mathcal{F}\|p) > \lambda\,. \tag{C26}$$

Evaluating (C25) on the set

$$\mathcal{Y}_{n,\zeta} := \left\{x^n :\ \frac{1}{2}\left\|t_{x^n} - \mathcal{F}_1\right\|_1 \leq \zeta\right\} = \bigcup_{t\in\mathcal{T}_n:\ \frac{1}{2}\|t-\mathcal{F}_1\|_1\leq\zeta} T_{n,t} \tag{C27}$$

of sequences whose type is ζ-close to a free probability distribution yields

$$\begin{aligned} q_n'\left(\mathcal{Y}_{n,\zeta}\right) &\leq 2^{n\lambda}\, p^{\otimes n}\left(\mathcal{Y}_{n,\zeta}\right) \\ &\overset{\text{(i)}}{\leq} 2^{n\lambda}\,(n+1)^{|\mathcal{X}|-1}\, 2^{-nD^{\zeta}(\mathcal{F}\|p)} \\ &\xrightarrow[n\to\infty]{\text{(ii)}} 0\,, \end{aligned} \tag{C28}$$

where in (i) we used Lemma 12, and (ii) follows from (C26).

We thus see that

$$q_n'\left(\mathcal{Y}_{n,\zeta}^c\right) \xrightarrow[n\to\infty]{} 1\,, \tag{C29}$$

entailing that

$$\liminf_{n\to\infty} q_n\left(\mathcal{Y}_{n,\zeta}^c\right) \geq 1-\varepsilon \tag{C30}$$

because of (C25). In particular,

$$q_n\left(\mathcal{Y}_{n,\zeta}^c\right) \geq \frac{1-\varepsilon}{2} \tag{C31}$$

for all sufficiently large n in the sequence. We further have

$$q_n\left(\mathcal{Y}_{n,\zeta}^c\right) = q_n\left(\bigcup\nolimits_{s\in\mathcal{T}_n:\ \frac{1}{2}\|s-\mathcal{F}_1\|_1>\zeta} T_{n,s}\right) = \sum_{s\in\mathcal{T}_n:\ \frac{1}{2}\|s-\mathcal{F}_1\|_1>\zeta} q_n(T_{n,s}) \tag{C32}$$

as well as $|\mathscr{T}_n| \leq (n+1)^{|\mathcal{X}|-1}$, and as such we can pick for all n in the sequence some $s_n \in \mathscr{T}_n$ such that $\frac{1}{2}\|s_n - \mathcal{F}_1\|_1 > \zeta$ and

$$q_n(T_{n,s_n}) \geq \frac{1-\varepsilon}{2\,(n+1)^{|\mathcal{X}|-1}}\,. \tag{C33}$$

By compactness, up to extracting a sub-sequence we can assume that $s_n \xrightarrow[n\to\infty]{} s$ for some $s \in \mathcal{P}(\mathcal{X})$. For the rest of this proof, unless otherwise specified, every limit $n \to \infty$ is understood to be taken on this sub-sequence. Note that $\frac{1}{2}\left\|s - \mathcal{F}_1\right\|_1 \geq \zeta$, implying in particular that

$$s \notin \mathcal{F}_1\,. \tag{C34}$$

Now, fix two arbitrarily small $\delta, \eta > 0$. By typicality,

$$s^{\otimes n}\left(\bigcup\nolimits_{t\in\mathscr{T}_n:\ \|s-t\|_\infty \leq \delta} T_{n,t}\right) \geq 1-\eta \tag{C35}$$

holds for all sufficiently large n. Therefore, the blurring lemma ensures that

$$\begin{aligned} D^\eta_{\max}\left(s^{\otimes n}\,\middle\|\,B_{n,m}(q_n)\right) &\overset{\text{(iii)}}{\leq} -\log_2 q_n\left(\bigcup\nolimits_{t\in\mathscr{T}_n:\ \|s-t\|_\infty\leq\delta} T_{n,t}\right) + ng\left(\left(2\delta+\tfrac{1}{n}\right)|\mathcal{X}|\right) \\ &\overset{\text{(iv)}}{\leq} -\log_2 q_n(T_{n,s_n}) + ng\left(\left(2\delta+\tfrac{1}{n}\right)|\mathcal{X}|\right) \\ &\overset{\text{(v)}}{\leq} -\log_2(1-\varepsilon) + 1 + (|\mathcal{X}|-1)\log_2(n+1) + ng\left(\left(2\delta+\tfrac{1}{n}\right)|\mathcal{X}|\right), \end{aligned} \tag{C36}$$

where $m = \lceil 2\delta n\rceil$. The justification of the above chain of inequalities is as follows: in (iii) we applied Lemma 9 with $p_n = s^{\otimes n}$ (which is clearly permutationally symmetric); (iv) holds for all sufficiently large n in the sub-sequence, because $s_n \xrightarrow[n\to\infty]{} s$; finally, in (v) we leveraged the estimate in (C33).

We are almost done. To conclude, it suffices to note that by Axioms 1, 2, 4, and 5 together with standard properties of the max-relative entropy from (C6) with $\varepsilon = 0$, it holds that

$$D_{\max}\left(B_{n,m}(q_n)\,\middle\|\,\mathcal{F}_n\right) \leq m|\mathcal{X}|\log_2\frac{1}{\mu} = \lceil 2\delta n\rceil\,|\mathcal{X}|\log_2\frac{1}{\mu}\,, \tag{C37}$$

where $\mu := \max_{q_0\in\mathcal{F}_1}\min_{x\in\mathcal{X}} q_0(x)$, which is strictly positive by Axiom 2. Therefore, combining (C36) and (C37) thanks to the triangle inequality for the smooth max-relative entropy [28], we see that

$$\begin{aligned} D^\eta_{\max}\left(s^{\otimes n}\,\middle\|\,\mathcal{F}_n\right) &\leq D^\eta_{\max}\left(s^{\otimes n}\,\middle\|\,B_{n,m}(q_n)\right) + D^\eta_{\max}\left(B_{n,m}(q_n)\,\middle\|\,\mathcal{F}_n\right) \\ &\leq -\log_2(1-\varepsilon) + 1 + (|\mathcal{X}|-1)\log_2(n+1) + ng\left(\left(2\delta+\tfrac{1}{n}\right)|\mathcal{X}|\right) + \lceil 2\delta n\rceil\,|\mathcal{X}|\log_2\frac{1}{\mu}\,. \end{aligned} \tag{C38}$$

We can now divide by n and take $n \to \infty$, which yields

$$\limsup_{n\to\infty}{}'\,\frac{1}{n}\,D^\eta_{\max}\left(s^{\otimes n}\,\middle\|\,\mathcal{F}_n\right) \leq g\left(2\delta|\mathcal{X}|\right) + 2\delta|\mathcal{X}|\log_2\frac{1}{\mu}\,, \tag{C39}$$

where we denoted with $\limsup'_{n\to\infty}$ the limit on the sub-sequence. Taking into account all values of $n \in \mathbb{N}^+$ instead of a sub-sequence only, the above reasoning shows that

$$\liminf_{n\to\infty}\frac{1}{n}\,D^\eta_{\max}\left(s^{\otimes n}\,\middle\|\,\mathcal{F}_n\right) \leq g\left(2\delta|\mathcal{X}|\right) + 2\delta|\mathcal{X}|\log_2\frac{1}{\mu}\,. \tag{C40}$$

Taking the limits $\eta \to 0^+$ and $\delta \to 0^+$ shows that

$$D^{\infty}(s\|\mathcal{F}) \overset{\text{(vi)}}{\leq} \lim_{\eta\to 0^+} \liminf_{n\to\infty} \frac{1}{n} D^{\eta}_{\max}\big(s^{\otimes n} \,\big\|\, \mathcal{F}_n\big) \leq 0\,, \tag{C41}$$

where (vi) is from asymptotic continuity. (Incidentally, Brandão–Plenio–Datta's asymptotic equipartition property [6, 29] guarantees that (vi) is actually an equality, although we are not using this particular fact here.) Since $D^{\infty}(s\|\mathcal{F}) \geq 0$ holds by construction, we have just proved that

$$D^{\infty}(s\|\mathcal{F}) = 0\,, \quad s \notin \mathcal{F}_1\,, \tag{C42}$$

which is in blatant contradiction with Axiom 6. ■

4. On the optimal test in the classical case

The difficult part of the proof of the generalised classical Sanov's theorem (Theorem 8), which has been presented in the above Section C 3, consists, in technical terms, in an achievability statement. Namely, by proving (C23) we have indirectly shown that there exists a tests that can distinguish $p^{\otimes n}$ from an arbitrary free probability distribution with asymptotically vanishing type II error and type I error exponent arbitrarily close to the reverse relative entropy $D(\mathcal{F}\|p)$. This naturally begs the question: how can such a test be designed in practice? Here we would like to argue that a test with this property can be described as follows. Given a string of alphabet symbols $x^n \in \mathcal{X}^n$, we start by calculating its type t_{x^n}, a probability distribution on $\mathcal{X}$ given by $t_{x^n}(x) := \frac{N(x|x^n)}{n}$, where $N(x|x^n)$ is the number of times x appears in x^n (see Section B 2). Then, for some small tolerance $\zeta > 0$:

- if $\frac{1}{2}\left\|t_{x^n} - \mathcal{F}_1\right\|_1 \leq \zeta$, then we guess that the underlying probability distribution is free;
- otherwise, we guess that it is p.

Using the notation introduced in Eq. (C27), an alternative way of rephrasing the above test is by saying that we guess p if and only if $x^n \notin \mathcal{Y}_{n,\zeta}$.

The type II error probability induced by the above test, i.e. the (worst-case) probability that a free probability distribution is mistakenly identified as p, is given by

$$\beta_n := \max_{q_n\in\mathcal{F}_n} \sum_{x^n\notin\mathcal{Y}_{n,\zeta}} q_n(x^n) = \max_{q_n\in\mathcal{F}_n} q_n\big(\mathcal{Y}^c_{n,\zeta}\big). \tag{C43}$$

The above proof shows that assuming Eq. (C30) for any diverging subsequence of values of n and for any $\varepsilon \in (0,1)$ leads to a contradiction, which is equivalent to stating that

$$\lim_{n\to\infty} \beta_n = 0\,. \tag{C44}$$

That is, the type II error probability vanishes asymptotically, which matches our first requirement for an optimal test.

What about the type I error exponent? The type I error probability α_n satisfies that

$$\alpha_n = \sum_{x^n\in\mathcal{Y}_{n,\zeta}} p^{\otimes n}(x^n) = p^{\otimes n}\big(\mathcal{Y}_{n,\zeta}\big) \leq 2^{-nD^{\zeta}(\mathcal{F}\|p)}, \tag{C45}$$

where the last inequality follows from Lemma 12, once one realises that the set $\left\{q \in \mathcal{P}(\mathcal{X}) : \frac{1}{2}\|q - \mathcal{F}_1\|_1 \leq \zeta\right\}$ is convex; here,

$$D^{\zeta}(\mathcal{F}\|p) := \min_{q:\, \frac{1}{2}\|q-\mathcal{F}_1\|_1 \leq \zeta} D(q\|p)\,. \tag{C46}$$

The relation (C45) shows directly that the type I error exponent is at least equal to $D^{\zeta}(\mathcal{F}\|p)$. By Lemma 10, $\lim_{\zeta\to 0^+} D^{\zeta}(\mathcal{F}\|p) = D(\mathcal{F}\|p)$; hence, by choosing $\zeta > 0$ sufficiently small, the exponent can be made arbitrarily close to the reverse relative entropy $D(\mathcal{F}\|p)$, meeting also the second requirement on the optimal test.

5. How to verify Axiom 6

Axiom 6 may seem like a strange one. For a start, it involves directly an entropic quantity, a feature that appears to be in contradiction with the philosophy of information theory, that prescribes that entropic quantities should find their meaning operationally rather than axiomatically. But even more worryingly, it may seem very hard to verify Axiom 6 for any given sequence of sets of probability distributions $(\mathcal{F}_n)_n$, as doing so would involve estimating a regularised quantity. To solve this issue, we illustrate here a simple sufficient condition that allows a swift verification of Axiom 6. The result below was proved by Piani in the fully quantum case in a pioneering work [7]. Here we need only its classical version, which we state here for the sake of completeness.

Lemma 13 [7, Theorem 1 and ensuing discussion]. *For a finite alphabet $\mathcal{X}$, let $(\mathcal{F}_n)_n$ be a sequence of sets of probability distributions $\mathcal{F}_n \subseteq \mathcal{P}(\mathcal{X}^n)$, and let $\mathbb{L} = (\mathbb{L}_n)_n$ be a sequence of sets of classical channels $\mathbb{L}_n$ on $\mathcal{X}^n$. If the compatibility condition in Definition 7 (see also (B16) for a classical formulation) is obeyed, then*

$$D^{\infty}(p\|\mathcal{F}) \geq D^{\mathbb{L}}(p\|\mathcal{F})\,; \tag{C47}$$

if, in addition, $\mathbb{L}_1$ is informationally complete and $\mathcal{F}$ is topologically closed, then $D^{\infty}(\cdot\|\mathcal{F})$ if faithful, in the sense that

$$D^{\infty}(p\|\mathcal{F}) > 0 \qquad \forall\, p \notin \mathcal{F}\,. \tag{C48}$$

Appendix D: Generalised quantum Sanov's theorem

To prove the result in the quantum case, we consider another axiom, concerned this time with the existence of a suitable informationally complete and compatible sequence of measurements. We will use this axiom to guarantee that Axiom 6 is satisfied for probability distributions resulting from this sequence, allowing us to apply the classical generalised Sanov's theorem (Theorem 8) and lift the classical result to the quantum case. Specifically, consider the following addition to the set of axioms studied in Section B 1.

6′. For some choice of numbers $r_n \in (0,1]$, the sequence $(\mathbb{M}_n)_n$ of sets of measurements

$$\mathbb{M}_n := \left\{ \left(\frac{\mathbb{1}^{\otimes n} + X_n}{2},\ \frac{\mathbb{1}^{\otimes n} - X_n}{2} \right) :\ X_n = X_n^{\dagger} \in \mathcal{L}\big(\mathcal{H}^{\otimes n}\big),\ \|X_n\|_{\infty} \leq r_n \right\}, \tag{D1}$$

where $\|\cdot\|_{\infty}$ denotes the operator norm, is compatible with $(\mathcal{F}_n)_n$.

We are now ready to state our main result in its most general form.

Theorem 14 (Generalised quantum Sanov's theorem). *Let $\mathcal{H}$ be a finite-dimensional Hilbert space, and let $(\mathcal{F}_n)_n$ be a sequence of sets of quantum states $\mathcal{F}_n \subseteq \mathcal{D}(\mathcal{H}^{\otimes n})$ that obeys the Brandão–Plenio axioms (Axioms 1–5 in Section B 1) as well as Axiom 6′. Then, we have that*

$$\lim_{n\to\infty} \frac{1}{n} D_H^{\varepsilon}\big(\mathcal{F}_n \,\big\|\, \rho^{\otimes n}\big) = D(\mathcal{F}\|\rho) \qquad \forall\, \rho \in \mathcal{D}(\mathcal{H}), \quad \forall\, \varepsilon \in (0,1)\,; \tag{D2}$$

in particular, the Sanov exponent is given by the single-letter expression

$$\mathrm{Sanov}(\rho\|\mathcal{F}) = D(\mathcal{F}\|\rho)\,. \tag{D3}$$

Before we present the proof of Theorem 14, it is instructive to see how it can be applied to calculate the Sanov exponent of entanglement testing. In what follows, $\mathcal{S}_{A:B}$ will denote the set of separable states [30] on the bipartite quantum system AB.

Corollary 15. *Let AB be a finite-dimensional bipartite quantum system. Then, it holds for all states ρ_{AB} that*

$$\lim_{n\to\infty} \frac{1}{n} D_H^{\varepsilon}\big(\mathcal{S}_{A^n:B^n} \,\big\|\, \rho_{AB}^{\otimes n}\big) = D\big(\mathcal{S}_{A:B} \,\big\|\, \rho_{AB}\big) = \inf_{\sigma_{AB}\in\mathcal{S}_{A:B}} D(\sigma_{AB}\|\rho_{AB}) \qquad \forall\, \varepsilon \in (0,1)\,. \tag{D4}$$

In particular, the Sanov exponent associated with entanglement testing, as well as the error exponent of entanglement distillation, are given by the single-letter formula

$$E_{d,\mathrm{err}}(\rho_{AB}) = \mathrm{Sanov}\big(\rho \,\big\|\, \mathcal{S}_{A:B}\big) = D\big(\mathcal{S}_{A:B} \,\big\|\, \rho_{AB}\big)\,. \tag{D5}$$

Here we note that our solution of the quantum Sanov's theorem in fact shows a strong converse property — the exponent does not depend on $\varepsilon \in (0,1)$ and uniformly equals $D\big(\mathcal{S}_{A:B} \,\big\|\, \rho_{AB}\big)$ for all ε. One immediate consequence of this is that our result for entanglement distillation is stronger than stated in the main text; namely, (D4) implies that

$$E_{d,\mathrm{err}}^{(m)}(\rho_{AB}) = D\big(\mathcal{S}_{A:B} \,\big\|\, \rho_{AB}\big) \qquad \forall m \in \mathbb{N}\,, \tag{D6}$$

where $E_{d,\mathrm{err}}^{(m)}$ is the m-copy distillation error exponent defined in (10). This shows that the distillation exponent does not actually depend on the target number of copies of the maximally entangled state at all.

Proof of Corollary 15. Setting $r_n = d^{-n/2}$, we see that for an arbitrary operator $X_n = X_n^\dagger \in \mathcal{L}\big(\mathcal{H}_{AB}^{\otimes n}\big)$ with $\|X_n\|_\infty \leq r_n$ we have that

$$\|X_n\|_2 \leq \sqrt{\dim\big(\mathcal{H}_{AB}^{\otimes n}\big)}\, \|X_n\|_\infty = d^{n/2}\, \|X_n\|_\infty \leq 1\,, \tag{D7}$$

implying, via [31, Theorem 1], that $\mathbb{1} \pm X_n \in \mathrm{cone}\big(\mathcal{S}_{A^n:B^n}\big)$. The compatibility between the set of measurements defined by (D1) and the set of separable states thus follows from the well-known compatibility between the sets of separable measurements and that of separable states [7, 17]. Axiom 6′ is therefore satisfied and we can thus immediately apply Theorem 14 to the case of entanglement testing. ∎

Remark 16. The compatibility assumption of Theorem 14 is quite versatile, and it can be shown to be obeyed by many resource theories of interest in quantum information and quantum computation. In all such cases, Theorem 14 provides us with the single-letter expression (D3) for the generalised Sanov exponent. Beyond the set of separable states, an exemplary case of this extension that is also relevant in the study of quantum entanglement is provided by the set of positive partial transpose (PPT) states $\mathcal{PPT}_{A:B}$ — see remark in Supplementary Note A and discussion of compatibility in [7, 17].

However, while these axiomatic assumptions can be verified for other sets of states relevant to quantum resource theories even beyond entanglement, it may not always be possible to establish an equivalence between the error exponent of resource testing and the error exponent of distillation as in Lemma 1. The study of other quantum resources for which this may be possible is an interesting open question.

Having illustrated its main consequences, we present now the full proof our main result, the generalised quantum Sanov's theorem.

Proof of Theorem 14. Given what we have already proved, it is not difficult to see that (D2) and (D3) are actually equivalent. In fact, due to (C10) and Proposition 11 we know that

$$\lim_{\varepsilon\to 1^-} \lim_{n\to\infty} \frac{1}{n} D_H^\varepsilon\big(\mathcal{F}_n \,\big\|\, \rho^{\otimes n}\big) = D(\mathcal{F}\|\rho) \qquad \forall\, \rho \in \mathcal{D}(\mathcal{H})\,. \tag{D8}$$

Since D_H^ε is monotonically non-decreasing in ε, to establish (D2) it thus suffices to prove that also

$$\mathrm{Sanov}(\rho\|\mathcal{F}) = \lim_{\varepsilon\to 0^+} \lim_{n\to\infty} \frac{1}{n} D_H^\varepsilon\big(\mathcal{F}_n \,\big\|\, \rho^{\otimes n}\big) \overset{?}{\geq} D(\mathcal{F}\|\rho) \qquad \forall\, \rho \in \mathcal{D}(\mathcal{H})\,. \tag{D9}$$

From now on, we will therefore focus on proving the above inequality.

Fix a positive integer $k \in \mathbb{N}^+$. Let $\mathbb{ALL}$ be the set of all quantum measurements with finitely many outcomes on any given quantum system. Since this set is closed under finite labelled mixtures, according to Lemma 6 we have that

$$D^{\mathbb{ALL}}\big(\mathcal{F}_k \,\big\|\, \rho^{\otimes k}\big) = \sup_{\mathcal{M}\in\mathbb{ALL}} \inf_{\sigma_k\in\mathcal{F}_k} D\big(\mathcal{M}(\sigma_k) \,\big\|\, \mathcal{M}\big(\rho^{\otimes k}\big)\big)\,. \tag{D10}$$

For some $\eta > 0$, let $\mathcal{M}_*$ be an almost optimal measurement for the supremum on the right-hand side of the above equation, i.e. let it satisfy

$$D\big(\mathcal{M}_*(\mathcal{F}_k) \,\big\|\, \mathcal{M}_*\big(\rho^{\otimes k}\big)\big) \geq \sup_{\mathcal{M}\in\mathbb{ALL}} \inf_{\sigma_k\in\mathcal{F}_k} D\big(\mathcal{M}(\sigma_k) \,\big\|\, \mathcal{M}\big(\rho^{\otimes k}\big)\big) - \eta = D^{\mathbb{ALL}}\big(\mathcal{F}_k \,\big\|\, \rho^{\otimes k}\big) - \eta\,. \tag{D11}$$

We denote by $(E_x)_{x\in\mathcal{X}}$ the POVM that represents $\mathcal{M}_*$. Without loss of generality, we can assume that $E_x \neq 0$ for all $x \in \mathcal{X}$.

We now have all the elements to explain our strategy to carry out hypothesis testing at the quantum level. We proceed as follows:

(1) We partition the n systems into $n' := \lfloor n/k \rfloor$ groups of k systems each, discarding the rest.

(2) We measure each group with the measurement $\mathcal{M}_*$, obtaining a sequence $x^{n'}$ of n' classical symbols $x_i \in \mathcal{X}$.

(3) We perform classical hypothesis testing on this sequence with the goal of minimising the type I error.

Provided that we can apply the generalised classical Sanov's theorem (Theorem 8), the above strategy gives the following estimate on the quantum Sanov exponent:

$$
\begin{aligned}
\mathrm{Sanov}(\rho\|\mathcal{F}) &\overset{(\star)}{\geq} \frac{1}{k} D\big(\mathcal{M}_*(\mathcal{F}_k)\,\big\|\,\mathcal{M}_*(\rho^{\otimes k})\big) \\
&\geq \frac{1}{k} D^{\mathbb{ALL}}\big(\mathcal{F}_k\,\big\|\,\rho^{\otimes k}\big) - \frac{\eta}{k} \\
&= \frac{1}{k} \inf_{\sigma_k\in\mathcal{F}_k} D^{\mathbb{ALL}}\big(\sigma_k\,\big\|\,\rho^{\otimes k}\big) - \frac{\eta}{k} \\
&\overset{(\mathrm{i})}{\geq} \frac{1}{k} \inf_{\sigma_k\in\mathcal{F}_k} D\big(\sigma_k\,\big\|\,\rho^{\otimes k}\big) - \frac{1}{k}\log_2\frac{(k+d-1)^{d-1}}{(d-1)!} - \frac{\eta}{k} \\
&\overset{(\mathrm{ii})}{=} D(\mathcal{F}\|\rho) - \frac{1}{k}\log_2\frac{(k+d-1)^{d-1}}{(d-1)!} - \frac{\eta}{k}\,.
\end{aligned}
\tag{D12}
$$

Here, $(\star)$ comes from the application of Theorem 8, whose legitimacy we will verify shortly. In (i) we used instead the entropic pinching inequality (B9), denoting with $d := \dim\mathcal{H}$ the dimension of $\mathcal{H}$, while (ii) follows by the additivity of the reverse relative entropy of resource. Taking the limit $k\to\infty$ of (D12) would thus yield precisely (D9) and therefore complete the proof.

We will now argue that the inequality $(\star)$ in (D12) holds, because we can legitimately apply Theorem 8. To this end, we construct the sequence of sets of probability distributions $\big(\widetilde{\mathcal{F}}_n\big)_n$ given by

$$
\widetilde{\mathcal{F}}_n := \mathcal{M}_*^{\otimes n}(\mathcal{F}_{nk}) \subseteq \mathcal{P}(\mathcal{X}^n)\,. \tag{D13}
$$

(We omit the dependence on k for simplicity.) If $(\mathcal{F}_n)_n$ satisfies the Brandão–Plenio axioms, as we have assumed, it is easy to verify that so does $\big(\widetilde{\mathcal{F}}_n\big)_n$. Let us verify this claim:

1. The convexity and closedness of $\widetilde{\mathcal{F}}_n$ follows from the convexity and closedness of $\mathcal{F}_{nk}$, due to the finite dimensionality and to the linearity of the copy-by-copy measurement map $\mathcal{M}_*^{\otimes n}$.

2. Due to Axiom 4 holding for $(\mathcal{F}_n)_n$, the set $\widetilde{\mathcal{F}}_1 = \mathcal{M}_*(\mathcal{F}_k)$ contains the probability distribution $q_0 := \mathcal{M}_*\big(\sigma_0^{\otimes k}\big)$, where $\sigma_0 \in \mathcal{F}_1$ is the full-rank state whose existence is guaranteed by Axiom 2. Note that $\sigma_0^{\otimes k}$ is full rank; thus, since $E_x \neq 0$ for all $x\in\mathcal{X}$ by assumption, it holds that $q_0(x) = \mathrm{Tr}\big[\sigma_0^{\otimes k}E_x\big] > 0$ for all $x\in\mathcal{X}$, i.e. q_0 has full support.

3. For a probability distribution $q = \mathcal{M}_*^{\otimes(n+1)}(\sigma) \in \widetilde{\mathcal{F}}_{n+1}$, where $\sigma\in\mathcal{F}_{(n+1)k}$, by repeated applications of Axiom 3 we see that tracing away the last system of q results in a probability distribution

$$
\mathrm{tr}_{n+1}\, q = \mathrm{tr}_{n+1}\,\mathcal{M}_*^{\otimes(n+1)}(\sigma) = \mathcal{M}_*^{\otimes n}\big(\mathrm{Tr}_{nk+1,\ldots,(n+1)k}\,\sigma\big) \in \mathcal{M}_*^{\otimes n}(\mathcal{F}_{nk}) = \widetilde{\mathcal{F}}_n\,. \tag{D14}
$$

4. If $q = \mathcal{M}_*^{\otimes n}(\sigma)\in\widetilde{\mathcal{F}}_n$ and $r = \mathcal{M}_*^{\otimes m}(\omega)\in\widetilde{\mathcal{F}}_m$, with $\sigma\in\mathcal{F}_{nk}$ and $\omega\in\mathcal{F}_{mk}$, then clearly

$$
q\otimes r = \mathcal{M}_*^{\otimes(n+m)}(\sigma\otimes\omega)\in\widetilde{\mathcal{F}}_{n+m} \tag{D15}
$$

simply because $\sigma\otimes\omega\in\mathcal{F}_{nk+mk} = \mathcal{F}_{(n+m)k}$. This establishes that $\big(\widetilde{\mathcal{F}}_n\big)_n$ is indeed closed under tensor products.

5. Given $q = \mathcal{M}_*^{\otimes n}(\sigma) \in \widetilde{\mathcal{F}}_n$ and some permutation $\pi \in S_n$, we will call

$$q_\pi(x^n) := q\left(\pi^{-1}(x^n)\right) = q\left(x_{\pi(1)} \dots x_{\pi(n)}\right) \tag{D16}$$

the permuted probability distribution. Then, denoting with $\widetilde{U}_\pi$ the unitary that implements π on each set of indices $\{i, i+k, \dots, i+(n-1)k\}$, where $i = 1, \dots, k$ (such a unitary is, of course, isomorphic to $U_\pi^{\otimes k}$, with U_π being the unitary that represents π on $\mathbb{C}^{\otimes n}$), we have that

$$\begin{aligned} q_\pi(x^n) &= q\left(x_{\pi(1)} \dots x_{\pi(n)}\right) \\ &= \mathrm{Tr}\left[\left(E_{\pi(x_1)} \otimes \dots \otimes E_{\pi(x_n)}\right) \sigma\right] \\ &= \mathrm{Tr}\left[\widetilde{U}_\pi^\dagger \left(E_{x_1} \otimes \dots \otimes E_{x_n}\right) \widetilde{U}_\pi\, \sigma\right] \\ &= \mathrm{Tr}\left[\left(E_{x_1} \otimes \dots \otimes E_{x_n}\right) \widetilde{U}_\pi \sigma \widetilde{U}_\pi^\dagger\right] \end{aligned} \tag{D17}$$

This shows that

$$q_\pi = \mathcal{M}_*^{\otimes n}\left(\widetilde{U}_\pi \sigma \widetilde{U}_\pi^\dagger\right) \in \mathcal{M}_*^{\otimes n}(\mathcal{F}_{nk}) = \widetilde{\mathcal{F}}_n\,, \tag{D18}$$

where we observed that $\widetilde{U}_\pi \sigma \widetilde{U}_\pi^\dagger \in \mathcal{F}_{nk}$ due to Axiom 5.

This proves that $\left(\widetilde{\mathcal{F}}_n\right)_n$ does indeed satisfy the Brandão–Plenio axioms. We now claim that the same family of sets satisfies also Axiom 6, because of our assumptions. To prove this claim, we will apply Piani's result in Lemma 13. To this end, we need to construct a sequence of sets of classical channels $\left(\mathbb{L}_n\right)_n$ that obeys the compatibility condition in Definition 7, re-written in (B16) for classical systems, and such that $\mathbb{L}_1$ is informationally complete, i.e. it satisfies (B15).

For each n and each sequence $\bar{x}^n \in \mathcal{X}^n$, consider the classical channel $\Lambda_{\bar{x}^n} : \mathbb{R}^{\mathcal{X}^n} \to \mathbb{R}^2$, whose output $y \in \{0,1\}$ is binary, defined by the conditional probability distribution

$$\Lambda_{\bar{x}^n}(y|x^n) := \frac{1}{2}\left(1 + (-1)^y r_{nk}\, \delta_{x^n, \bar{x}^n}\right), \tag{D19}$$

where $r_{nk} \in (0,1]$ is the number appearing in (D1), and $\delta_{x^n,\bar{x}^n} := \prod_{i=1}^n \delta_{x_i, \bar{x}_i}$, with δ representing the Kronecker delta. We now construct the sequence $\left(\mathbb{L}_n\right)_n$ of sets of classical channels

$$\mathbb{L}_n := \{\Lambda_{\bar{x}^n} :\ \bar{x}^n \in \mathcal{X}^n\}\,. \tag{D20}$$

Note that $\mathbb{L}_1$ is informationally complete according to (B15), because

$$\mathrm{span}\left\{(\Lambda_{\bar{x}}(y|x))_x :\ \bar{x} \in \mathcal{X},\ y \in \{0,1\}\right\} = \mathbb{R}^{\mathcal{X}}. \tag{D21}$$

To verify the above identity, it suffices to consider a vector $v \in \mathbb{R}^{\mathcal{X}}$ that is orthogonal to the set on the left-hand side, i.e. it satisfies

$$0 = 2\sum_x v_x\, \Lambda_{\bar{x}}(y|x) = \sum_x v_x + (-1)^y r_k v_{\bar{x}} \qquad \forall\, \bar{x} \in \mathcal{X}, \quad \forall\, y \in \{0,1\}\,. \tag{D22}$$

Summing over y we obtain that $\sum_x v_x = 0$, which plugged into the above identity shows that $v = 0$. This proves (D21), establishing that $\mathbb{L}_1$ is indeed informationally complete.

We now set out to verify the compatibility condition (B16). Consider the free probability distribution

$$q_{n+m} = \mathcal{M}_*^{\otimes(n+m)}\left(\sigma_{(n+m)k}\right) \in \widetilde{\mathcal{F}}_{n+m}\,. \tag{D23}$$

For some $\bar{x}^n \in \mathcal{X}^n$ and $\tilde{x}^m \in \mathcal{X}^m$, we construct

$$\begin{aligned} \tilde{q}_m(\tilde{x}^m) &:= \sum_{x^n} \Lambda_{\bar{x}^n}(y|x^n)\, q_{n+m}(x^n \tilde{x}^m) \\ &= \sum_{x^n} \Lambda_{\bar{x}^n}(y|x^n)\, \mathrm{Tr}\left[\left(E_{x_1}\otimes\ldots\otimes E_{x_n}\otimes E_{\tilde{x}_1}\otimes\ldots\otimes E_{\tilde{x}_m}\right)\sigma_{(n+m)k}\right] \\ &= \mathrm{Tr}\left[\left(E_{\tilde{x}_1}\otimes\ldots\otimes E_{\tilde{x}_m}\right)\mathrm{Tr}_{1,\ldots,nk}\left[\left(\left(\sum\nolimits_{x^n}\Lambda_{\bar{x}^n}(y|x^n)\, E_{x_1}\otimes\ldots\otimes E_{x_n}\right)\otimes \mathbb{1}^{\otimes mk}\right)\sigma_{(n+m)k}\right]\right] \\ &\overset{\text{(iii)}}{=} \frac{1}{2}\,\mathrm{Tr}\left[\left(E_{\tilde{x}_1}\otimes\ldots\otimes E_{\tilde{x}_m}\right)\mathrm{Tr}_{1,\ldots,nk}\left[\left(\left(\mathbb{1}^{\otimes nk} + (-1)^y r_{nk}\, E_{\bar{x}_1}\otimes\ldots\otimes E_{\bar{x}_n}\right)\otimes \mathbb{1}^{\otimes mk}\right)\sigma_{(n+m)k}\right]\right]. \end{aligned} \tag{D24}$$

In (iii) we used the definition (D19) and observed that

$$\sum_{x^n} E_{x_1}\otimes\ldots\otimes E_{x_n} = \left(\sum\nolimits_x E_x\right)^{\otimes n} = \mathbb{1}^{\otimes nk}. \tag{D25}$$

due to the normalisation condition for the POVM $(E_x)_x$. The calculation in (D24) proves that

$$\begin{aligned} \tilde{q}_m &= \mathcal{M}_*^{\otimes m}\left(\mathrm{Tr}_{1,\ldots,nk}\left[\left(\left(\frac{\mathbb{1}^{\otimes nk} + (-1)^y r_{nk}\, E_{\bar{x}_1}\otimes\ldots\otimes E_{\bar{x}_n}}{2}\right)\otimes \mathbb{1}^{\otimes mk}\right)\sigma_{(n+m)k}\right]\right) \\ &\overset{\text{(iv)}}{\in} \mathcal{M}_*^{\otimes m}\left(\mathrm{cone}(\mathcal{F}_{mk})\right) \\ &= \mathrm{cone}\left(\mathcal{M}_*^{\otimes m}(\mathcal{F}_{mk})\right) \\ &= \mathrm{cone}\left(\widetilde{\mathcal{F}}_m\right), \end{aligned} \tag{D26}$$

which proves that the sets of probability distributions $\left(\widetilde{\mathcal{F}}_n\right)_n$ and the sets of classical channels $\left(\mathbb{L}_n\right)_n$ satisfy the compatibility conditions in (B16). In the above calculation, (iv) follows from our assumption that the set of measurements in (D1) is compatible (in the quantum sense of Definition 7) with the sets of free states $(\mathcal{F}_n)_n$, because

$$\left\|(-1)^y r_{nk}\, E_{\bar{x}_1}\otimes\ldots\otimes E_{\bar{x}_n}\right\|_\infty = r_{nk}\left\|E_{\bar{x}_1}\right\|_\infty\cdots\left\|E_{\bar{x}_n}\right\|_\infty \le r_{nk}\,. \tag{D27}$$

This concludes the proof that $\left(\widetilde{\mathcal{F}}_n\right)_n$ satisfies not only the Brandão–Plenio axioms, but also Axiom 6 in Section B 1. This means that the application of the generalised classical Sanov's theorem (Theorem 8) marked as (⋆) in (D12) is justified. According to the above discussion, taking the limit $k \to \infty$ in (D12) completes the proof. ■

Similarly to what we did in Section C 4 for the classical case, also in the quantum case we can ask ourselves how to design a sequence of tests that achieves the optimal performance advertised in Theorem 14. Namely, for any given ρ, the tests should discriminate $\rho^{\otimes n}$ from an arbitrary free state $\sigma_n \in \mathcal{F}_n$, while exhibiting:

(a) a vanishing type II error probability; and

(b) a type I error exponent arbitrarily close to the reverse relative entropy $D(\mathcal{F}\|\rho)$.

To construct such a test, one can proceed as follows. Denoting by ε an overall tolerance on the final type I error exponent, we start by choosing some $\eta > 0$ and some integer $k \in \mathbb{N}^+$ such that the last two terms on the rightmost side of (D12) are smaller than $\varepsilon/2$. This is certainly possible, as the sum of the two vanishes in the limit where $k \to \infty$ (here d, the underlying Hilbert space

dimension, is fixed). We now need to find a measurement $\mathcal{M}_*$ that satisfies (D11); since η and k have been fixed, and thus all involved states have bounded dimension, this can be accomplished numerically. (The complexity of this step, naturally, will depend on the precise nature of the sets of free states $\mathcal{F}_k$, e.g. on whether they admit an efficient description.)

We can now proceed as described in the above proof: we divide the n quantum systems into $n' = \lfloor n/k \rfloor$ groups of k copies each, discarding the remainder, and measure each group with $\mathcal{M}_*$, obtaining a string of classical symbols $x^{n'}$. To this string we can apply the classically optimal test designed in Section C 4, which consists in ascertaining whether $\frac{1}{2}\left\|t_{x^{n'}} - \mathcal{M}_*(\mathcal{F}_k)\right\|_1 \leq \zeta$, where $\zeta > 0$ is a small parameter, or not; in the first case, we guess that the underlying state was free, while in the second we guess that it was $\rho^{\otimes n}$. As explained in Section C 4, the type II error probability associated with this test vanishes asymptotically as $n \to \infty$ (and thus $n' \to \infty$). The overall type I error exponent is at least

$$\frac{1}{k}\, D^{\zeta}\big(\mathcal{M}_*(\mathcal{F}_k)\,\big\|\,\mathcal{M}_*(\rho^{\otimes k})\big) \geq \frac{1}{k}\, D\big(\mathcal{M}_*(\mathcal{F}_k)\,\big\|\,\mathcal{M}_k(\rho^{\otimes k})\big) - \frac{\varepsilon}{2} \geq D(\mathcal{F}\|\rho) - \varepsilon\,, \tag{D28}$$

where the first inequality holds due to Lemma 10, provided that we choose $\zeta > 0$ to be sufficiently small, while the second holds by construction, due to the choice of k.

Appendix E: Further considerations

1. Additivity violation for the reverse Rényi relative entropy of entanglement

We now set out to show that the Rényi-α reverse relative entropy of entanglement is never weakly additive for $\alpha < 1$. To this end, it suffices to take as a counterexample the antisymmetric Werner state ρ_1 [30, 32], humorously referred to as the 'universal counterexample in entanglement theory' [33]. Consider the bipartite states on $\mathbb{C}^d \otimes \mathbb{C}^d$ given by

$$\rho_a := \frac{\mathbb{1} + (-1)^a F}{d(d + (-1)^a)}\,, \qquad a = 0,1\,, \qquad F := \sum_{i,j=1}^{d} |ij\rangle\!\langle ij|\, ji\,; \tag{E1}$$

incidentally, these are the extremal points of the one-parameter family of Werner states $\rho(\delta) := (1-\delta)\rho_0 + \delta\rho_1$, with $\delta \in [0,1]$ [30]. In what follows, we will omit the dependence on d for simplicity.

Lemma 17. *Let $\alpha \in (0,1)$, and let $\mathbb{D}_\alpha$ be any quantum divergence that reduces to the Rényi-α divergence for classical (i.e. commuting) states and obeys the data processing inequality. Then the corresponding reverse relative entropy of entanglement, defined by $\mathbb{D}_\alpha(\mathcal{S}\|\rho) := \min_{\sigma\in\mathcal{S}} \mathbb{D}_\alpha(\sigma\|\rho)$, fails to be (weakly) additive on many copies of the antisymmetric state ρ_1, in formula*

$$\mathbb{D}_\alpha\big(\mathcal{S}\,\big\|\,\rho_1^{\otimes 2}\big) < 2\,\mathbb{D}_\alpha(\mathcal{S}\|\rho_1)\,. \tag{E2}$$

If $\mathbb{D}_\alpha$ is also assumed to be additive, then the claim of the above lemma can be seen as a consequence of the results in [34, Section 5]. To see why, recall that there it is argued that any function of the form $\rho \mapsto \min_{\sigma\in\mathcal{S}} \mathbb{D}(\rho\|\sigma)$, where $\mathbb{D}$ is a function on pairs of quantum states that obeys data processing, additivity, and normalisation — meaning that $\mathbb{D}\big(|0\rangle\!\langle 0|\,\big\|\,\frac{1}{2}(|0\rangle\!\langle 0| + |1\rangle\!\langle 1|)\big) = 1$ — fails to be additive on two copies of the antisymmetric state. Taking $\mathbb{D}(\rho\|\sigma) = \frac{1-\alpha}{\alpha}\,\mathbb{D}_\alpha(\sigma\|\rho)$, where $\mathbb{D}_\alpha$ is a divergence as in the statement of Lemma 17, yields $\min_{\sigma\in\mathcal{S}} \mathbb{D}(\rho\|\sigma) = \frac{1-\alpha}{\alpha}\,\mathbb{D}_\alpha(\mathcal{S}\|\rho)$; assuming additivity, one then deduces the claim of Lemma 17. (This observation

was communicated to us by R. Rubboli after the first version of our manuscript appeared [35].) Below, we present our independent proof of Lemma 17, as it is short, rather direct, and it by-passes the additivity requirement.

Proof of Lemma 17. It is well known, via symmetry arguments (see, e.g., the discussion around [36, Eq. (4)]), that the closest separable state to $\rho(\delta)^{\otimes n}$, for any $\delta \in [0,1]$, is of the form

$$\sigma = \sum_{a_1,\ldots,a_n \in \{0,1\}^n} P_{a_1,\ldots,a_n}\, \rho_{a_1} \otimes \ldots \rho_{a_n}\,, \tag{E3}$$

where P is an arbitrary probability distribution on $\{0,1\}^n$. This is usually argued for the standard relative entropy of entanglement, with the optimisation with respect to separable states on the second argument, but it holds equally well for $\mathbb{D}_\alpha(\mathcal{S}\|\rho)$ — in fact, the proof only uses data processing and the fact that the Werner twirling map is separability preserving.

Now, for a state σ of the form in (E3) we have that

$$\mathbb{D}_\alpha\big(\sigma \,\big\|\, \rho_1^{\otimes n}\big) \overset{\text{(i)}}{=} D_\alpha(P \,\|\, e_{1^n}) \overset{\text{(ii)}}{=} -\frac{\alpha}{1-\alpha}\log_2 P_{1^n} \overset{\text{(iii)}}{=} \frac{\alpha}{1-\alpha} D\big(\rho_1^{\otimes n} \,\big\|\, \sigma\big)\,. \tag{E4}$$

Here, in (i) we made use of the fact that $\mathbb{D}_\alpha$ must reduce to its classical version when the states commute — and σ and $\rho_1^{\otimes n}$ do commute — in (ii) we introduced the probability distribution e_{1^n} defined by $e_{1^n}(x^n) = \prod_{i=1}^n \delta_{x_i,1}$, where $\delta_{a,b}$ is the Kronecker delta, and finally in (iii) we considered the *standard* relative entropy, with σ in the second argument. Optimising over separable states σ of the form in (E3) we obtain that

$$\mathbb{D}_\alpha\big(\mathcal{S} \,\big\|\, \rho_1^{\otimes n}\big) = \frac{\alpha}{1-\alpha} D\big(\rho_1^{\otimes n} \,\big\|\, \mathcal{S}\big)\,. \tag{E5}$$

Since it is well known that $D\big(\rho_1^{\otimes 2} \,\big\|\, \mathcal{S}\big) < 2\,D(\rho_1\|\mathcal{S})$ [37], the proof is complete. ■

2. A (classical) counter-example

Here, we construct a set of classical probability distribution that obeys the Brandão–Plenio Axioms 1 – 5 but whose Sanov exponent is different from the reverse relative entropy. In other words, this shows that the Brandão–Plenio axioms do not suffice by themselves to guarantee that the generalized Sanov exponent is given by the reverse relative entropy. At the root of this discrepancy is the fact that in this model the exponent, i.e. the regularised relative entropy, can vanish even for resourceful states.

Consider a device that outputs a symbol $x \in \{0,1\}$ every time it is used. What you would really like it to do is to output the symbol 1 many times. Unfortunately, all that it does is that it sets itself in a random state 0 or 1, outputs the corresponding symbol some unknown number of times, and then it resets again in a random 0/1 state (as struck by a lightning, or a cosmic ray), and it continues in that way until it has produced a total of n symbols. You then get all the symbols it produced, but shuffled in some random order. The corresponding set of probability distributions is given by

$$\mathcal{F}_n := \operatorname{conv}\left\{ r_{J_1} \otimes \ldots \otimes r_{J_k} :\ J_1,\ldots,J_k \text{ is a partition of } [n] \right\}, \tag{E6}$$

where

$$r_J\big(x^J\big) := \begin{cases} 1/2 & \text{if } x^J = 0^J \text{ or } x^J = 1^J, \\ 0 & \text{otherwise.} \end{cases} \tag{E7}$$

Lemma 18. *The family of sets $\mathcal{F}_n$ satisfies the Brandão–Plenio Axioms 1 – 5.*

Proof. Convex, closedness, and permutation invariance are clear from the definition. Closure under tensorisation follows from the fact that joining a partition of $[n]$ and one of $[m]$ yields a partition of $[n+m]$. The uniform probability distribution r_1 on $\{0,1\}$ is in $\mathcal{F}_1$ and has full support. It only remains to verify closure under partial trace. This is also easy, and it follows from the observation that $\mathrm{tr}_k\, r_k = r_{k-1}$ for all $k \geq 2$, where tr_k denotes the operation of discarding the last symbol. ■

We are now ready to present our counter-example. Consider the probability distribution e_1 with the property that $e_1(0) = 0$ and $e_1(1) = 1$. Clearly, $e_1 \notin \mathcal{F}_1$ is not a free distribution — after all, it corresponds to what we would like the device to do, not to what it does in reality. However,

$$D\big(e_1^{\otimes n} \,\big\|\, \mathcal{F}_n\big) \leq D_{\max}\big(e_1^{\otimes n} \,\big\|\, \mathcal{F}_n\big) \leq D_{\max}(e_1^{\otimes n} \| r_n) = 1\,, \tag{E8}$$

implying that $D^\infty(e_1\|\mathcal{F}) = 0$.

What about the reverse quantities? Rather trivially, $D(\mathcal{F}\|e_1) = +\infty$ because e_1 is a deterministic probability distribution which is not in $\mathcal{F}_1$. The same happens (as it should, because the reverse relative entropy is additive) for n copies. What is even more interesting is that one can evaluate the hypothesis testing relative entropy $\min_{q_n\in\mathcal{F}_n} D_H^\varepsilon(q_n\|e_1^{\otimes n})$ explicitly. Indeed, the best test $a : \{0,1\}^n \to [0,1]$ satisfies $a(x^n) = 1$ for $x^n \neq 1^n$; its only un-specified entry, $a(1^n)$, can be determined by requiring that

$$1 - \varepsilon \leq \sum_{x^n} a(x^n) q_n(x^n) = a(1^n) q_n(1^n) + 1 - q_n(1^n)\,, \tag{E9}$$

which immediately gives us $a(1^n) = \left(1 - \frac{\varepsilon}{q_n(1^n)}\right)_+$. This in turn shows that

$$D_H^\varepsilon\big(q_n \,\big\|\, e_1^{\otimes n}\big) = -\log_2 \sum_{x^n} a(x^n)\, e_1^{\otimes n}(x^n) = -\log_2 a(1^n) = \begin{cases} -\log_2\left(1 - \frac{\varepsilon}{q_n(1^n)}\right) & \text{if } \varepsilon < q_n(1^n), \\ +\infty & \text{otherwise,} \end{cases} \tag{E10}$$

Since this is a non-increasing function of $q_n(1^n)$, it is minimised when this number is as large as possible, which happens when $q_n = r_n$, in which case it evaluates to $1/2$. Hence,

$$\min_{q_n\in\mathcal{F}_n} D_H^\varepsilon\big(q_n \,\big\|\, e_1^{\otimes n}\big) = D_H^\varepsilon\big(r_n \,\big\|\, e_1^{\otimes n}\big) = \begin{cases} -\log_2(1-2\varepsilon) & \text{if } \varepsilon < 1/2, \\ +\infty & \varepsilon \geq 1/2. \end{cases} \tag{E11}$$

In particular,

$$\liminf_{n\to\infty} \frac{1}{n} \min_{q_n\in\mathcal{F}_n} D_H^\varepsilon\big(q_n \,\big\|\, e_1^{\otimes n}\big) = \begin{cases} 0 & \text{if } \varepsilon < 1/2, \\ +\infty & \varepsilon \geq 1/2, \end{cases} \tag{E12}$$

entailing that

$$\mathrm{Sanov}(e_1\|\mathcal{F}) = \lim_{\varepsilon\to 0^+} \liminf_{n\to\infty} \frac{1}{n} \min_{q_n\in\mathcal{F}_n} D_H^\varepsilon\big(q_n \,\big\|\, e_1^{\otimes n}\big) = 0 < \infty = D(\mathcal{F}\|e_1)\,. \tag{E13}$$

For completeness, we also consider the Stein setting. It is straightforward to verify that

$$D_H^\varepsilon\big(e_1^{\otimes n} \,\big\|\, q_n\big) = -\log_2(1-\varepsilon) - \log_2 q_n(1^n)\,, \tag{E14}$$

so that $\min_{q_n\in\mathcal{F}_n} D_H^{\varepsilon}\big(e_1^{\otimes n}\,\big\|\,q_n\big) = -\log_2(1-\varepsilon) - 1$, implying that

$$\liminf_{n\to\infty} \frac{1}{n} \min_{q_n\in\mathcal{F}_n} D_H^{\varepsilon}\big(e_1^{\otimes n}\,\big\|\,q_n\big) = 0 \qquad \forall\, \varepsilon \in [0,1)\,, \tag{E15}$$

and in turn that $\mathrm{Stein}(e_1\|\mathcal{F}) = 0 = D^{\infty}(e_1\|\mathcal{F})$.

---

[1] J. v. Neumann, *Zur Theorie der Gesellschaftsspiele*, Math. Ann. **100**, 295 (1928).
[2] H. Nagaoka, *The converse part of the theorem for quantum Hoeffding bound*, arXiv:quant-ph/0611289 (2006).
[3] M. Horodecki, P. Horodecki, and R. Horodecki, *General teleportation channel, singlet fraction, and quasidistillation*, Phys. Rev. A **60**, 1888 (1999).
[4] G. Vidal and R. Tarrach, *Robustness of entanglement*, Phys. Rev. A **59**, 141 (1999).
[5] Z. Lin, private communication (2024).
[6] F. G. S. L. Brandão and M. B. Plenio, *A generalization of quantum Stein's lemma*, Commun. Math. Phys. **295**, 791 (2010).
[7] M. Piani, *Relative entropy of entanglement and restricted measurements*, Phys. Rev. Lett. **103**, 160504 (2009).
[8] M. Fekete, *Über die Verteilung der Wurzeln bei gewissen algebraischen Gleichungen mit ganzzahligen Koeffizienten*, Math. Z. **17**, 228 (1923).
[9] M. J. Donald, *On the relative entropy*, Commun. Math. Phys. **105**, 13 (1986).
[10] D. Petz, *Sufficient subalgebras and the relative entropy of states of a von Neumann algebra*, Commun. Math. Phys. **105**, 123 (1986).
[11] M. Berta, O. Fawzi, and M. Tomamichel, *On variational expressions for quantum relative entropies*, Lett. Math. Phys. **107**, 2239 (2017).
[12] M. Hayashi, *Optimal sequence of quantum measurements in the sense of Stein's lemma in quantum hypothesis testing*, J. Phys. A **35**, 10759 (2002).
[13] M. Tomamichel, *Quantum Information Processing with Finite Resources: Mathematical Foundations* (Springer, 2015).
[14] M. Berta, F. G. S. L. Brandão, and C. Hirche, *On composite quantum hypothesis testing*, Commun. Math. Phys. **385**, 55 (2021).
[15] D. Sutter, M. Berta, and M. Tomamichel, *Multivariate trace inequalities*, Commun. Math. Phys. **352**, 37 (2017).
[16] F. Hiai and D. Petz, *The proper formula for relative entropy and its asymptotics in quantum probability*, Comm. Math. Phys. **143**, 99 (1991).
[17] F. G. S. L. Brandão, A. W. Harrow, J. R. Lee, and Y. Peres, *Adversarial hypothesis testing and a quantum Stein's lemma for restricted measurements*, IEEE Trans. Inf. Theory **66**, 5037 (2020).
[18] R. Renner, *Security of quantum key distribution*, Ph.D. thesis, ETH Zurich (2005).
[19] N. Datta, *Min- and max-relative entropies and a new entanglement monotone*, IEEE Trans. Inf. Theory **55**, 2816 (2009).
[20] L. Lami, *A Solution of the Generalized Quantum Stein's Lemma*, IEEE Trans. Inf. Theory **71**, 4454 (2025).
[21] M. Tomamichel and M. Hayashi, *A hierarchy of information quantities for finite block length analysis of quantum tasks*, IEEE Trans. Inf. Theory **59**, 7693 (2013).
[22] A. Anshu, M. Berta, R. Jain, and M. Tomamichel, *A minimax approach to one-shot entropy inequalities*, J. Math. Phys. **60**, 122201 (2019).
[23] B. Regula, L. Lami, and N. Datta, *Tight relations and equivalences between smooth relative entropies*, arXiv:2501.12447 (2025).
[24] M. Tomamichel, R. Colbeck, and R. Renner, *A Fully Quantum Asymptotic Equipartition Property*, IEEE Trans. Inf. Theory **55**, 5840 (2009).
[25] I. Sanov, *On the probability of large deviations of random variables*, Mat. Sbornik **42**, 11 (1957).
[26] T. M. Cover and J. A. Thomas, *Elements of Information Theory*, Wiley Series in Telecommunications and Signal Processing (Wiley-Interscience, New York, NY, USA, 2006).
[27] I. Csiszár and J. Körner, *Information theory: coding theorems for discrete memoryless systems*, 2nd ed., Probability and Mathematical Statistics (Cambridge University Press, Cambdridge, UK, 2011).

[28] M. Christandl and A. Müller-Hermes, *Relative entropy bounds on quantum, private and repeater capacities*, Commun. Math. Phys. **353**, 821 (2017).

[29] N. Datta, *Max-relative entropy of entanglement, alias log robustness*, Int. J. Quantum Inf. **07**, 475 (2009).

[30] R. F. Werner, *Quantum states with Einstein-Podolsky-Rosen correlations admitting a hidden-variable model*, Phys. Rev. A **40**, 4277 (1989).

[31] L. Gurvits and H. Barnum, *Separable balls around the maximally mixed multipartite quantum states*, Phys. Rev. A **68**, 042312 (2003).

[32] M. Christandl, N. Schuch, and A. Winter, *Entanglement of the antisymmetric state*, Commun. Math. Phys. **311**, 397 (2012).

[33] S. Aaronson, S. Beigi, A. Drucker, B. Fefferman, and P. Shor, *The power of unentanglement*, in *Proc. 23rd IEEE Annual Conf. Comput. Complex.*, CCC '08 (IEEE Computer Society, USA, 2008) pp. 223–236.

[34] R. Rubboli and M. Tomamichel, *New Additivity Properties of the Relative Entropy of Entanglement and Its Generalizations*, Commun. Math. Phys. **405**, 162 (2024).

[35] R. Rubboli, private communication (2024).

[36] K. Audenaert, J. Eisert, E. Jané, M. B. Plenio, S. Virmani, and B. De Moor, *Asymptotic relative entropy of entanglement*, Phys. Rev. Lett. **87**, 217902 (2001).

[37] K. G. H. Vollbrecht and R. F. Werner, *Entanglement measures under symmetry*, Phys. Rev. A **64**, 062307 (2001).
